# Lessons from Red Data Books: Plant Vulnerability Increases with Floral Complexity

**DOI:** 10.1371/journal.pone.0138414

**Published:** 2015-09-21

**Authors:** Anastasia Stefanaki, Aphrodite Kantsa, Thomas Tscheulin, Martha Charitonidou, Theodora Petanidou

**Affiliations:** 1 Laboratory of Biogeography and Ecology, Department of Geography, University of the Aegean, Mytilene, Greece; 2 Naturalis Biodiversity Center – National Herbarium of the Netherlands, Leiden, Netherlands; University of Northampton, UNITED KINGDOM

## Abstract

The architectural complexity of flower structures (hereafter referred to as floral complexity) may be linked to pollination by specialized pollinators that can increase the probability of successful seed set. As plant—pollinator systems become fragile, a loss of such specialized pollinators could presumably result in an increased likelihood of pollination failure. This is an issue likely to be particularly evident in plants that are currently rare. Using a novel index describing floral complexity we explored whether this aspect of the structure of flowers could be used to predict vulnerability of plant species to extinction. To do this we defined plant vulnerability using the Red Data Book of Rare and Threatened Plants of Greece, a Mediterranean biodiversity hotspot. We also tested whether other intrinsic (e.g. life form, asexual reproduction) or extrinsic (e.g. habitat, altitude, range-restrictedness) factors could affect plant vulnerability. We found that plants with high floral complexity scores were significantly more likely to be vulnerable to extinction. Among all the floral complexity components only floral symmetry was found to have a significant effect, with radial-flower plants appearing to be less vulnerable. Life form was also a predictor of vulnerability, with woody perennial plants having significantly lower risk of extinction. Among the extrinsic factors, both habitat and maximum range were significantly associated with plant vulnerability (coastal plants and narrow-ranged plants are more likely to face higher risk). Although extrinsic and in particular anthropogenic factors determine plant extinction risk, intrinsic traits can indicate a plant’s proneness to vulnerability. This raises the potential threat of declining global pollinator diversity interacting with floral complexity to increase the vulnerability of individual plant species. There is potential scope for using plant—pollinator specializations to identify plant species particularly at risk and so target conservation efforts towards them.

## Introduction

The criteria for the designation of endangered species, as established worldwide by the International Union for the Conservation of Nature (IUCN), are based on trends in population sizes and geographical ranges [1]. These data are enriched with information on other external threats, mostly anthropogenic, including overexploitation, habitat degradation, pollutants, and competition from introduced species [1]. Various efforts have been made to complement the IUCN criteria (e.g. [2–4]), which have focused primarily on geographical and/or ecological factors, with endemism or habitat often cited as reliable predictors of plant vulnerability (e.g. [2]). It is, however, possible that other intrinsic factors, including the flowering duration, reproduction or pollination mode of plants, may affect their vulnerability [5]. Incomplete knowledge of the reproduction biology of many rare and threatened plants [6, 7] impedes the consideration of such intrinsic traits associated with the reproductive success of plants.

A determinant of successful plant reproduction is pollination. A complex flower structure may increase a plant’s chances of successful reproduction by enhancing pollinator fidelity to flowers and reducing larceny by illegitimate floral visitors ([8] and references therein). On the other hand, plants with complex flowers may be likely to suffer from pollination failure, if their handling requires too specialized pollinators that are rare or for some reason (e.g. human impact) become lost, or if they depend on large-sized pollinators that are naturally less abundant or on pollinators with fluctuating populations over space and time. Particularly for rare plants, pollination failure may be critical, because these plants are more likely to be pollen limited due to co-flowering species competing for limited pollinator numbers [9, 10]. The vulnerability of plant species caused by the lack of pollinators has been long discussed through the prism of the plants’ interspecific competition for shared pollinators ([11] and references therein, [12–14]). The importance of this problem becomes even more relevant as the concern for human-induced breakdown of plant—pollinator systems increases [15–21].

Here we investigate whether plant vulnerability can be predicted by floral structure (simple to complex) vis-à-vis other potential predictors of vulnerability, using as a case example the rare and threatened plants of Greece. With 6600 plant taxa (1072 genera, 185 families) Greece is regarded as a hotspot for biodiversity and endemism within the Mediterranean [22, 23]. A large number of these taxa (1462, i.e. 22.15% of the flora) are considered endemic to Greece including numerous narrow endemics restricted to a single mountain or island [23]. The endangered Greek flora is described in two editions of the Red Data Books of Rare and Threatened Plants of Greece (hereafter Greek Red Data Book). The first was published in 1995 with accounts of 263 taxa [24]. A two-volume book based on the current IUCN criteria [1] was published in 2009 as complementary to that of 1995 and included updates of some previous accounts plus accounts of many additional taxa [25]. In total, the number of plant taxa included in the Greek Red Data Book corresponds to *c*. 7% of the Greek flora, while half of the country’s endemic, rare and threatened plants have not been evaluated [26].

This study addresses the following questions: (1) Are plants with complex flower structures more vulnerable than those with simpler flower structures? (2) Are other intrinsic factors associated with plant vulnerability, such as pollination-related (flowering times, floral color and size) and life strategy ones (life form, capability of asexual reproduction)? (3) What is the effect of extrinsic factors (e.g. geographical, ecological) on plant vulnerability? Floral complexity was quantified using the Floral Complexity Index introduced here, i.e. an index taking into account several floral traits related to presumed pollinator specialization. We discuss the effects of floral complexity along with other factors shown here to affect plant vulnerability and address the necessity of joint plant—pollinator conservation assessments.

## Materials and Methods

### Dataset

A dataset of intrinsic and extrinsic variables (described in detail below) was generated for the plant taxa included in the Greek Red Data Book [24, 25]. The latter was the main source of information for the compilation of the dataset with supplementary sources comprising major floristic works [23, 27–34], online Herbarium collections (mainly from the Herbaria B, E, K, LD, MAIC and W), original descriptions of taxa and other taxonomic and floristic papers, books and online databases [35–58]. Nomenclature was updated according to APG III [59] for plant families and according to Dimopoulos et al. [23] for species and subspecies considering synonymies and excluding erroneous records as reported in [23]. Wind- and water- pollinated taxa were also excluded from the dataset, which finally included a total of 427 plant taxa (S1 Table), all pollinated by insects, the only pollinators in the Mediterranean area.

### Intrinsic variables

#### Floral complexity variables and Floral Complexity Index

We used several floral variables shown in the literature to be functionally important for pollination, which we presumed to be involved in plants' selectivity for pollinators and thus related to plant vulnerability. These were (1) shape, (2) depth, (3) symmetry, (4) corolla segmentation, and (5) functional reproductive unit. An additional approach involved the assembling of these variables into a floral complexity index described below.

Floral shape. According to the functional shape of flowers (or of flower aggregations functioning as single attraction units, see functional reproductive unit below), taxa were assigned to 11 levels based on the classification by Fægri and van der Pijl [60], Barth [61] and Petanidou [62]. These levels were: (1) bell (a downward facing bell-shaped flower that the insect enters with much of or the entire body, not only proboscis); (2) brush (a single flower or a flower aggregation with numerous protruding anthers); (3) disk (a shallow flower with petals more or less spread out in a flat circle); (4) tube (a tubular flower); (5) disk-tube (a flower with a flattened part abruptly arising on a tubular stalk); (6) funnel (an upward facing funnel-shaped flower that the insect enters with much of or the entire body); (7) flag (the “butterfly”-shaped flower of the Fabaceae and Polygalaceae); (8) gullet (a flower with a lip serving as a landing platform for insects to insert their head or whole body into the corolla tube); (9) head (a densely-packed flower aggregation with more or less spherical or flat appearance; (10) lip (an orchid flower with an extended lip used by visiting insects as a landing platform); and (11) trap flowers (a bowl or more complicated tubular structure with steep and smooth surface where the insects get “trapped” for some period of time).

Floral depth. Floral depth, measured as length of the corolla tube, expresses the accessibility of nectar reward accumulated in the bottom of the flower tube to a flower visitor standing at the free surface of the flower. Plant taxa were assigned to three levels, viz. those having: (1) low-depth flowers, i.e. with corolla tube length < 4 mm, including those without floral depth (disk, brush flowers); (2) medium-depth flowers, with corolla tube length 4–10 mm; and (3) high-depth flowers, with corolla tube length >10 mm [62]. Particularly in flag flowers the depth was expressed as the length of the keel, in trap flowers (Araceae) as the length of the spathe tube, and in spurred flowers (e.g. taxa of Violaceae, Fumariaceae, Ranunculaceae, Orchidaceae) as the length of the spur.

Floral symmetry. Taxa were assigned to two levels based on the number of floral symmetry axes, viz. those having: (1) several axes (radial symmetry); and (2) one axis (bilateral symmetry). Floral symmetry in Asteraceae was estimated for the whole inflorescence (see functional reproduction unit below).

Corolla segmentation. Three degrees of corolla (or perianth) segmentation were considered: (1) sympetaly, when petals (or perianth segments) are fused along their entire length; (2) choripetaly, when petals (or perianth segments) are completely free; and (3) semichoripetaly, when petals (or perianth segments) are fused to some degree, forming a more or less distinct corolla tube with free lobes. Calyx segmentation was not considered.

Functional reproductive unit. This corresponds to the inflorescence and includes flower aggregations from a functional rather than a morphological (plant taxonomy) viewpoint [62]. Taxa were assigned accordingly to three levels, described as having: (1) single flowers, including any type of inflorescence with up to five concurrently functional (open) flowers; (2) aggregations of flowers arising on a flat or spherical surface of the plant (heads, umbels, corymbs); and (3) aggregations of flowers arising along a cylindrical surface of the plant (spikes, racemes, panicles).

The Floral Complexity Index (FCI henceforth) considers floral complexity as the plants' “selectivity” for pollinators and is based on the five floral variables described above. Each variable was given a weight (*w*) based on its potential contribution to floral complexity: high for floral shape (w_shape_ = 0.3); moderate for depth, symmetry and corolla segmentation (w_depth_ = w_symmetry_ = w_corolla segmentation_ = 0.2); and low for functional reproductive unit (w_functional reproductive unit_ = 0.1). The sum of the five weights equals 1. Each level of the variables depth, symmetry, corolla segmentation and functional reproductive unit was assigned a value (*V*) based on a scale from 1 to 3, where 1 indicates a less specialized trait and 3 a more specialized trait. For floral shape, due to greater number of levels (i.e. shape types of different levels of difficulty), values (*V*) were assigned based on a scale from 1 to 5. Each variable level’s value (*V*) was the mean of assigned scores by four pollination experts (see S1 Text). The final weight of each variable defining its contribution to the index was then calculated by multiplying the variable’s weight (*w*) by the variable level’s value (*V*) (Table 1). The FCI for each taxon was finally expressed as the sum of final weights of the five variables: *FCI* = ∑(*w*_*j*_ * *V*_*ij*_), where *w*_*j*_ = the weight of the floral variable *j* and *V*_*ij*_ = the value of the floral variable *j* for taxon *i*. For example, the FCI value of a taxon with racemes of gullet-shaped flowers (i.e. bilaterally symmetrical and partly fused) and a long (>10 mm) floral tube would be the sum of these final weights: FCI = 0.30 + 1.13 + 0.60 + 0.40 + 0.60 = 3.03 (see Table 1). The FCI value assigned to each taxon of the dataset is given in S1 Table.

**Table 1:** Weights of the floral variables used in the Floral Complexity Index. For details on the estimation of the variable level values see S1 Text. All terms are explained in the text.

Floral variable (*w*: variable weight)	Level or trait	Level value (*V*)^a^	Final weight^b^
**Shape (*w* = 0.3)**	bell	3.33	1.00
	brush	3.50	1.05
	disk	1.00	0.30
	tube	3.25	0.98
	disk-tube	2.75	0.83
	funnel	2.65	0.80
	flag	4.25	1.28
	gullet	3.75	1.13
	head	2.25	0.68
	lip	4.50	1.35
	trap	4.25	1.28
**Depth (*w* = 0.2)**	low-depth	1.00	0.20
	medium-depth	2.00	0.40
	high-depth	3.00	0.60
**Symmetry (*w* = 0.2)**	bilateral	3.00	0.60
	radial	1.13	0.23
**Corolla segmentation (*w* = 0.2)**	sympetalous	2.50	0.50
	semichoripetalous	2.00	0.40
	choripetalous	1.50	0.30
**Functional reproductive unit (w = 0.1)**	single	1.50	0.15
	spikes/racemes	3.00	0.30
	heads	1.50	0.15

^a^Based on a scale of 1–3 (for floral depth, symmetry, corolla segmentation and functional reproductive unit) or a scale of 1–5 (for floral shape). See S1 Text.

^b^Resulting from the floral variable weight (*w*) multiplied by the variable level’s value (*V*).

#### Other intrinsic variables

Floral color. Five levels were adopted according to Petanidou and Lamborn [63]: (1) white; (2) yellow; (3) violet, purple, pink, red; (4) blue; and (5) green. In cases of two or more concurrent colors, the dominant (> 50% of flower surface) was selected, and in taxa with individuals bearing flowers of different colors the most frequent was taken.

Floral size. This was expressed as the length—width average of the exposed surface of the corolla in a 2D-projection (corresponding e.g. to the floral diameter for more or less circular flowers). Taxa were assigned to three levels: (1) small, 1–10mm; (2) medium, 10–20mm; and (3) large, >20mm [62]. In cases of strongly compact inflorescences, which give the “impression of functionally single flowers”, e.g. heads and compound corymbs of Asteraceae or compound umbels of Apiaceae, floral size was measured for the whole inflorescence.

Flowering season. Three flowering seasons were considered: (1) February to May; (2) June to September; and (3) October to January. When the flowering time of a taxon fell into two different seasons it was assigned to the dominant one (biggest overlap). If a taxon fell equally in two seasons it was assigned to the earliest.

Flowering duration. The total number of months during which a taxon has been reported to be in flower.

Life form. Taxa were assigned to four groups according to Petanidou et al. [64]: (1) therophytes, i.e. annual plants that survive unfavorable seasons in the form of seeds; (2) geophytes, i.e. perennial plants that survive unfavorable seasons in the form of underground storage organs (bulbs, tubers, corms, rhizomes); (3) herbaceous perennials, i.e. perennial plants that are entirely herbaceous above the ground or only woody at the base; and (4) woody perennials, i.e. perennial plants more or less entirely woody.

Asexual reproduction. Taxa were classified as known or not known to reproduce by vegetative means, e.g. bulbs, tubers, corms, rhizomes, stolons, suckers.

### Extrinsic variables

#### Habitat

Taxa were assigned according to the habitats they occur in. This was based on habitat associations described in Dimopoulos et al. [23] as: (1) aquatic (freshwater) habitats; (2) cliffs and other rocky habitats; (3) lowland to montane grasslands; (4) high-mountain vegetation; (5) coastal and marine habitats; (6) phrygana (low scrubland); (7) agricultural and ruderal habitats; and (8) woodlands and scrub. When more than one habitat was provided for a taxon, the dominant habitat according to the Red Data Book description was retained.

#### Minimum altitude

This denotes the minimum altitude (m), where a taxon has been recorded.

#### Maximal distance

A quantitative measure of range, denoting the Euclidean distance (in km) between the most distant localities of a taxon as provided in the Red Data Book dot maps. For its measurement we digitized the maps from the Red Data Book in ArcGIS 9.3 (ESRI), considering also recent updates in distributions of the taxa by Dimopoulos et al. [23].

#### Range-restricted status

According to this qualitative measure of range introduced by Dimopoulos et al. [23], taxa were assigned to two levels: (1) range-restricted, i.e. taxa with a distribution area whose longest dimension does not exceed 500 km [23]; and (2) not range-restricted. This variable was selected instead of endemism as non-affected by country borders.

#### Phytogeographical region

Taxa were assigned to the three phytogeographical regions of Greece *sensu* Brummitt [65]: (1) Kriti—Karpathos group; (2) East Aegean Islands; and (3) remaining Greece. When a taxon fell into more than one region, it was assigned to the one with the highest number of localities it was recorded in. If a taxon occurred in equal numbers of localities in more than one region, its extent of occurrence (*sensu* IUCN [1]) in each region was additionally estimated and the taxon was consequently assigned to the region in which its extent of occurrence is larger.

### Data analysis

The possible outcomes of the response variable “plant vulnerability” were expressed by the IUCN categories as given for each taxon in the Greek Red Data Book, updated where applicable according to the IUCN Red List of Threatened Species (www.iucnredlist.org) and aggregated in two groups: (1)“Less threatened”, including Vulnerable (VU), Near Threatened (NT), Rare (R) and Least Concerned (LC) taxa; and (2) “More threatened”, including Critically Endangered (CR), Endangered (EN) and presumed Extinct (EX) taxa. In contrast to the IUCN approach [1], i.e. assembling VU together with CR and EN taxa in a “threatened” group, we adopted a stricter distinction among “less threatened” and “more threatened” taxa, with VU taxa placed in the first group. This was also necessary in order to avoid potential inconsistency in our dataset that might arise from the presence of the old IUCN category “R” of the plants drawn from the 1995 Red Data Book [24]: when pulling the data out of both Red Data Books [24, 25] we realized that assigning a taxon as R in [24] occasionally appeared to overlap with that for VU as currently formed in [1] and as used in the 2009 Red Data Book [25].

A Generalized Linear Model (GLM) was fitted in R 3.1.1 [66] using the ‘glm’ function with a binomial probability distribution, in order to explore the relation of plant vulnerability to the independent intrinsic and extrinsic variables (main effects). A stepwise backward model selection procedure using the Akaike Information Criterion (AIC) was performed to select the best model. The final model was constructed on the basis of the AIC. As is the case with binary traits in logistic GLM, goodness-of-fit in the final model was tested graphically with validation plots of the empirical probabilities with their standard errors [67]. All independent variables were checked for collinearity before regression analysis. In order to test for the effects of each level of the categorical independent variables on plant vulnerability, we ran the ‘glm’ function for each level separately and calculated the significance of their effects with the likelihood ratio chi-square tests of the deviance analysis.

In order to check for the potential effects of the components of the FCI (viz. floral depth, floral shape, floral symmetry, functional reproductive unit, and corolla segmentation), we conducted the following steps: We ran (1) bivariate tests where the effects of each one of the single floral complexity components and of FCI itself were tested individually (S2 Text); (2) a full GLM model with all individual intrinsic variables including floral complexity components (viz. floral depth, floral shape, floral symmetry, functional reproductive unit, corolla segmentation, floral color, floral size, flowering duration, flowering season, life form and asexual reproduction) and the extrinsic ones (viz. habitat, minimum altitude, maximal distance, range-restricted status and phytogeographical region) (S2 Text); (3) a GLM model with each single floral complexity component and all other intrinsic and extrinsic variables (S2 Text); (4) the GLM with the floral complexity components replaced by the FCI itself in the full model.

**Fig 1:**
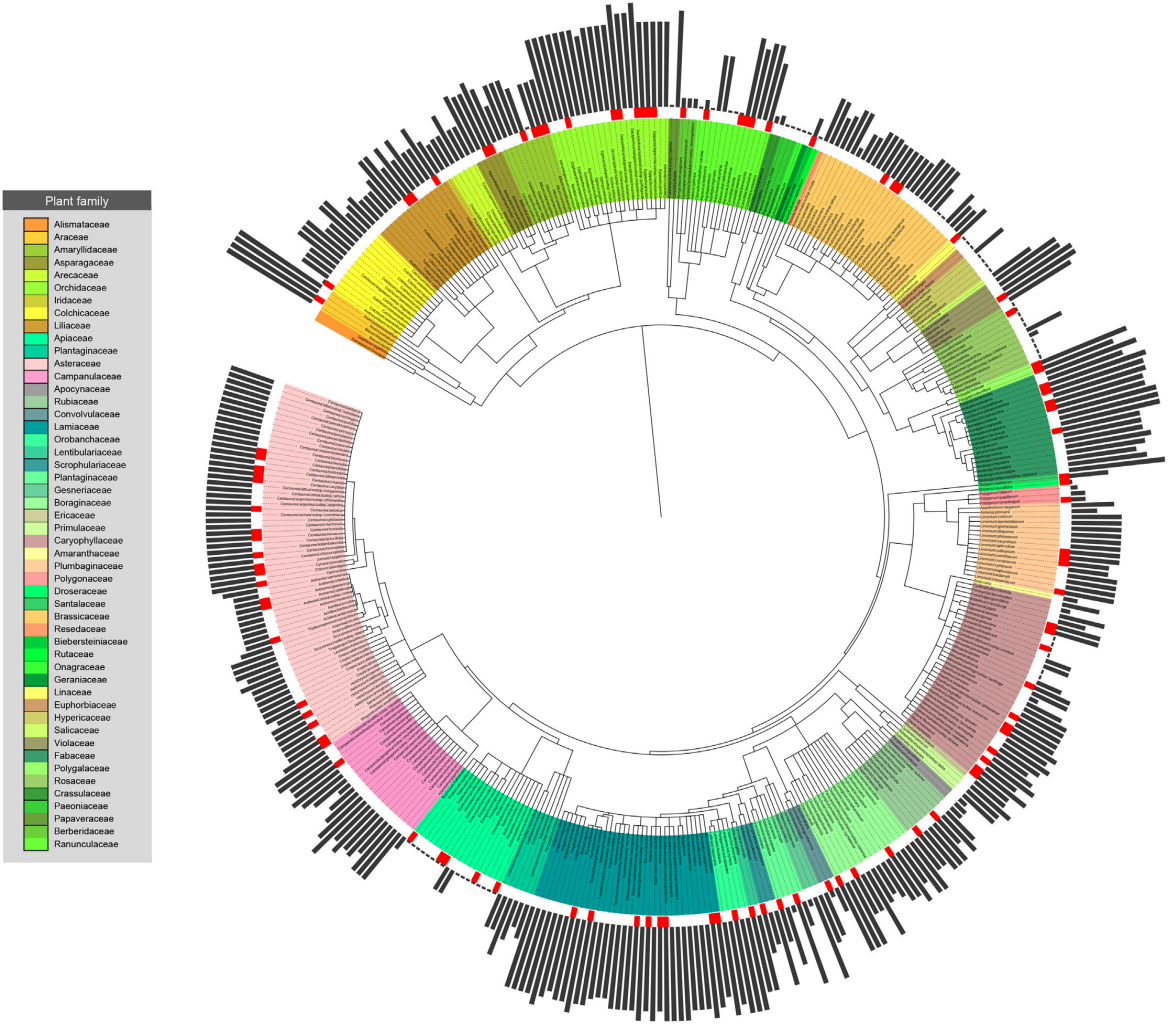
The Floral Complexity Index (FCI) values distributed across the Greek rare and threatened plants’ phylogeny. Grey bars indicate the relative magnitude of the FCI (highest value: 3.25, lowest: 1.15). Red rectangles mark the “more threatened” (CR, EN or EX) taxa.

Before choosing our standard non-phylogenetic statistical modeling approach, we tested our dataset for phylogenetic dependence regarding the vulnerability of the plants. The phylogeny of the 427 plants of our dataset was constructed according to APG III [59] using the online software Phylomatic v3 [68]. The branch lengths of this phylogenetic tree were adjusted so as to correspond to evolutionary divergence time between clades, using the ‘bladj’ algorithm in the software Phylocom v4.2 [69]. For this calibration, we used the updated node ages provided by Gastauer and Meira-Neto [70], which address the inconsistencies observed in the default ages file of Phylocom based on Wikstrom’s dating of Angiosperm families [71]. A phylogenetic tree (Fig 1) was created using the web-based Interactive Tree of Life v2 [72]. Both the raw response variable and its residuals in the final model were tested for phylogenetic signal (see [73] and references therein). To estimate the phylogenetic signal, i.e. to test whether plant vulnerability follows a random phylogenetic pattern or it is phylogenetically clumped corresponding to a Brownian motion model, we measured (i) for the raw variable, the *D* statistic for binary traits, which is based on the sum of the sister-clade differences of the given trait in a phylogeny [74] using the ‘phylo.D’ function in the R package caper 0.5.2 and (ii) for the residuals, both Blomberg’s K [75] and Pagel’s λ [76] using functions ‘phylosignal’ in the R package picante 1.6–2 and ‘pgls’ (by fitting the model: vulnerability~1) in caper 0.5.2. All metrics revealed no significant phylogenetic signal of plant vulnerability (see S3 Text), allowing us to proceed with the above-mentioned GLM approach.

## Results

### Floral Complexity Index

The FCI scores for the 427 taxa examined varied between 1.15 and 3.25. The lowest score (1.15) was recorded for several plants in eight families (e.g. Papaveraceae, Geraniaceae, Paeoniaceae), while the highest (>2.50) for plants of Araceae, Fumariaceae, Orobanchaceae, Fabaceae, Orchidaceae, Lamiaceae, Polygalaceae, Lentibulariacae and Violaceae. Two subspecies of the orchid *Dactylorhiza kalopissii*, subsp. *kalopissii* and subsp. *pythagorae*, designated, respectively, as Endangered and Critically Endangered, obtained the highest complexity score of 3.25 (Fig 1; S1 Table).

### Plant vulnerability

Bivariate tests for the effects of each of the components of the FCI showed that no single floral complexity component (viz. floral shape, floral depth, floral symmetry, corolla segmentation, functional reproductive unit) is a source of variation for plant vulnerability (S2 Text). When components were individually entered in the full GLM including all other intrinsic (viz. floral color, floral size, flowering duration, flowering season, life form, asexual reproduction) and extrinsic factors (viz. habitat, minimum altitude, maximal distance, range-restricted status, phytogeographical region), we found separate statistically significant effects only for two components, i.e. floral symmetry and functional reproductive unit. When all single floral complexity components were entered together in the full model, only floral symmetry showed a significant effect (S2 Text). More specifically, taxa with radially symmetrical (actinomorphic) flowers showed a negative correlation to vulnerability (vulnerability ~ radial floral symmetry estimate: -0.86, *z*-statistic = -2.95, *p*-value = 0.003). Consequently, each dimension of floral complexity alone does not seem to be a predictor of plant vulnerability, and floral symmetry is the strongest predictor of all the components when added to the multivariate model. Lastly, we compared two full models including either symmetry or FCI plus all other intrinsic and extrinsic factors mentioned above; we found that the model including FCI performed better than the model including symmetry based on AIC and the Bayesian Information Criterion (S2 Text).

Our finally tested GLM included FCI plus all other intrinsic and extrinsic factors as independent variables. Based on the AIC stepwise backward model selection the best fitting model included FCI, life form and floral color among the intrinsic factors; and maximal distance, habitat and minimum altitude among the extrinsic ones (Table 2). The variables asexual reproduction, flowering season, flowering duration, floral size, phytogeographical region and range-restricted status were non-significant and thus discarded from the final model based on the AIC.

**Table 2:** Results of the best fitting (based on AIC) GLM showing the effects of the intrinsic and extrinsic variables on the Greek rare and threatened plants’ vulnerability.

Independent variables		LR *χ* ^*2*^	Df	*P*-value
**Intrinsic**	Life form	10.183	3	0.0171
	Floral color	8.602	4	0.0719
	Floral Complexity Index	10.230	1	0.0014
**Extrinsic**	Habitat	18.129	7	0.0114
	Minimum altitude	3.012	1	0.0826
	Maximal distance	12.683	1	0.0004

**Fig 2:**
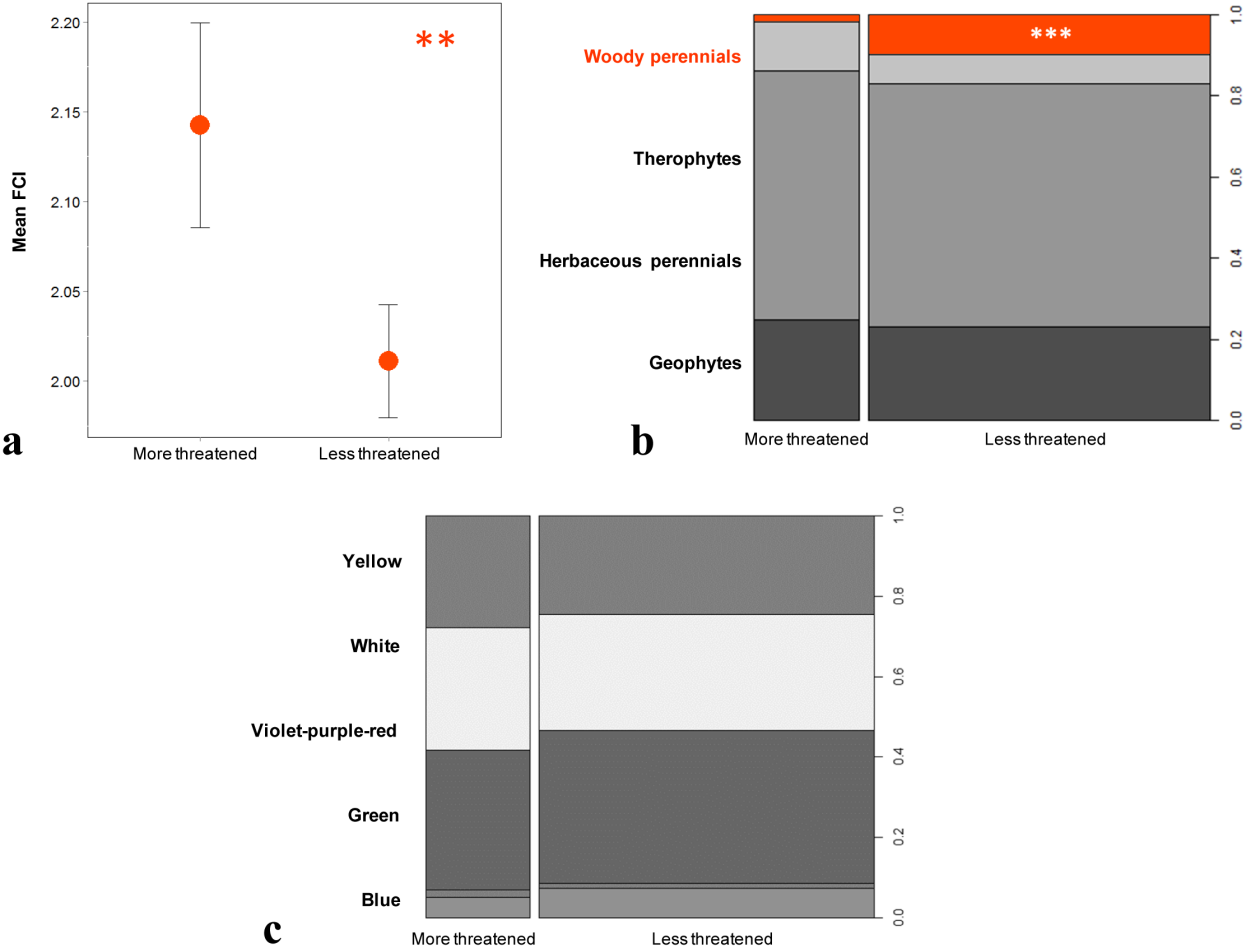
Intrinsic vulnerability factors of the Greek rare and threatened plants. Effects of the intrinsic variables on plant vulnerability as included in the best fitted logistic GLM based on the backward AIC selection process. (a) Mean value of the FCI (±SE); (b) life form; and (c) floral color. Categorical independent variables (b, c) are presented in spinograms. The width of the columns corresponds to the relative frequency of the “more threatened” and “less threatened” plants in the dataset; the heights of the cells represent the relative frequency of the response variable in every level of the explanatory variables. Colored cells denote statistical significance of the respective level (**: ≤ 0.01, ***: ≤ 0.001).

**Fig 3:**
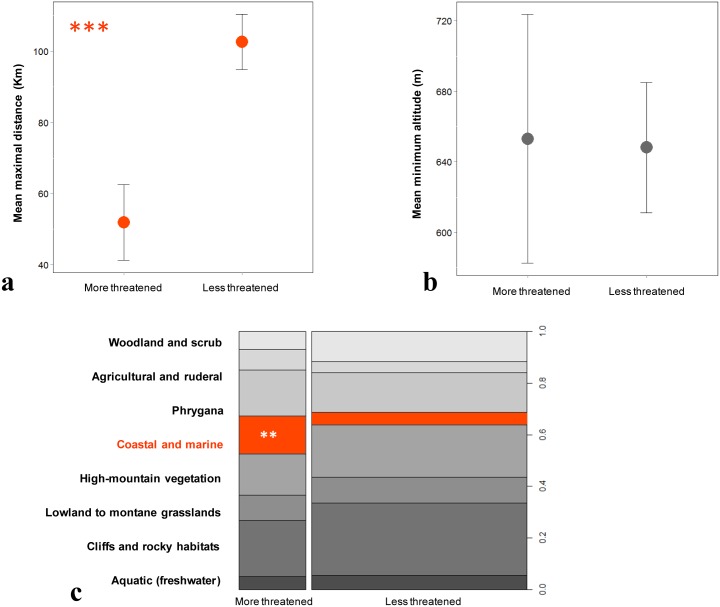
Extrinsic vulnerability factors of the Greek rare and threatened plants. The effects of the extrinsic variables on plant vulnerability as included in the best fitted logistic GLM based on the backward AIC selection process. (a) Mean maximal distance (±SE); (b) mean minimum altitude (±SE); and (c) habitat. The variation of the latter is presented in a spinogram. The width of the columns corresponds to the relative frequency of the “more threatened” and “less threatened” plants in the dataset; the height of the cells represents the relative frequency of the response variable in every type of habitat. Colored cells denote statistical significance of the respective level (**: ≤ 0.01, ***: ≤ 0.001).

The mean FCI was found to be significantly higher in the “more threatened” than the “less threatened” taxa (Fig 2a). Both these plant groups are characterized by the high proportion of herbaceous perennials and geophytes but differ significantly in the proportion of woody perennials (higher in the “less threatened”); the presence of therophytes is slightly higher in the “more threatened” than in the “less threatened” group, albeit without significant difference (Fig 2b). Among all variables examined, maximal distance between a taxon’s most distant populations was found to be the most significant (Table 2) with this measure of maximum range being smallest in the “more threatened” compared to the “less threatened” plants (Fig 3a). Both plant groups occur in all habitat categories, with the “more threatened” being significantly more frequent in coastal habitats. “More threatened” taxa tend to be more frequent in ruderal habitats, whereas “less threatened” are more likely to be encountered in cliffs, woodland/scrub and high-mountain habitats—although these differences were not statistically significant (Fig 3c). Floral color and minimum altitude, although present in the best model, have p-values marginally outside the significance level (Table 2) and so do not differ between the two plant groups (Figs 2c and 3b).

## Discussion

### Effects of intrinsic factors on plant vulnerability

Among all floral variables examined (viz. FCI, floral size, floral color, flowering duration and season), the FCI was found to be the most predictive for a taxon’s vulnerability: taxa with higher floral complexity are more likely to be more threatened. A possible explanation for this may be pollination limitation; such limitation may be due to specialized pollinator scarcity or to the limited attraction by the small population sizes threatened plants normally occur in [16, 77].

A key role may be attributed to floral symmetry, which was the only one among FCI components having per se a significant effect on plant vulnerability. The “more threatened” plants examined here were found to have more frequently bilaterally symmetrical flowers (44.3% of the “more threatened” plants) compared to the “less threatened” (28%). Bilateral floral symmetry is suggested to provide multiple advantages for a plant, such as higher visitation, more efficient recognition and faster handling by pollinators and higher outcrossing rates than radial symmetry [78–80]. Moreover, in a community context floral symmetry has been shown to predict differences in pollinator visitation within a plant—pollinator network [81]. However, bilateral symmetry might also turn into a disadvantage in times that populations of associated specialized pollinators decline [78]. Further than floral symmetry having an important effect on plant vulnerability, the higher significance of floral complexity vs. that of its individual components implies that flower complexity is a more meaningful predictor, and when considered together not only the symmetry but also the shape and depth of the flowers, their segmentation and their aggregation in inflorescences are influential for plant vulnerability. Evidence from empirical studies points towards the same direction. For example, it has been found that sympetalous tubular (deep) flowers, whose handling requires pollinators with long mouth parts, receive fewer insect visits than open choripetalous flowers [82], the latter being more commonly visited by insects that are less specialized [83]. Likewise, plant species with complex floral shape (e.g. flag or gullet) were shown to depend on specialized pollinator behavior compared to species with less complex flowers (e.g. disk or head), which have a more generalized pollination system [82]. Finally, the organization of floral reproductive units at different levels of aggregations (e.g. simple or dense inflorescences) also affects pollinator visitation in different ways: on the one hand increased inflorescence size or inflorescence flower density may enhance floral attractiveness to pollinators, but on the other it may add to the difficulty of the inflorescence’s handling on the pollinator’s part [62, 84, 85].

Our results did not reveal any particular color to be linked with any of the two plant vulnerability groups examined. In fact, the proportions of colors within both the “more threatened” and “less threatened” plants are similar to the patterns recorded in Mediterranean and worldwide floras ([63] and references therein). However, due to a very weak association of floral color with plant vulnerability further investigation may be required, ideally considering several other parameters in addition to the dominant colors examined here—e.g. brightness, intensity, contrast or color patterns and UV clues [62, 63, 86].

The significantly higher frequency of woody perennials among the “less threatened” plants recorded here implies that woody perennial plant forms are confronted with lower threat risk. This is in agreement with earlier findings [87]. Perhaps the resistance against threats is related to particular characteristics that woody perennials have, such as the perennial habit, which can safeguard population dynamics over the long term. On the other hand, the overrepresentation of therophytes (annuals) in the “more threatened” vs. the “less threatened” group, even though not significant, suggests that the unstable dynamics and population fluctuations associated with the annual life cycle may place such plants at greater risk (see also similar findings by Fenu et al. [88]).

The absence of significance of asexual reproduction for plant vulnerability in our study is interesting nevertheless not totally surprising. Although asexual reproduction is regarded as a reproduction mode that may compensate for pollination failure, allowing rare species to reproduce where seed production is not possible, it cannot guarantee long-term survival of rare plants due to gradual loss of genetic diversity where a persistent decline in pollination occurs [16, 89]. This contrast between the short-term advantages and long-term disadvantages of asexual reproduction may underlie the context-dependence of this plant trait as suggested by Godefroid et al. [90], who found asexual reproduction to be associated on the one hand with plant decline, but on the other with plant expansion in different regions’ floras.

### Effects of extrinsic factors on plant vulnerability

Among the extrinsic factors examined, the most interesting was habitat, an “envelope variable” encompassing the human factor as a plant vulnerability driver through land-use change, which is the dominant threat reported for the Greek rare and threatened plants [91]. Indeed, we found that plants growing in habitats with severe human pressure, i.e. coastal and ruderal/agricultural, are more threatened than those growing on cliffs or high-mountain vegetation, which are typically less affected by human activity. Coastal habitats are in particular more threatened in the Mediterranean [22], with similar results being reported from Sardinia, where again, cliffs tend to be rich in rare plants (i.e. “less threatened” in our study) compared to plants in coastal habitats (sand dunes), which are the most threatened [2].

In conservation biology, rarity indicators, such as endemism, are fundamental in setting conservation priorities. Interestingly, range restrictedness, i.e. the alternative estimation of endemism examined in this study, was not found to be significantly related to plant vulnerability. However, it is possible that should a smaller threshold be used to define range-restrictedness than the 500 km adopted here (see e.g. [92]), this factor may have been more meaningful. This complies also with the high significance in our results of maximal distance (i.e. the distance in km between a taxon’s most distant populations), its continuous numerical nature making it a more accurate rarity estimate.

Although altitude was shown here to be associated with plant vulnerability, no clear differences could be observed between the more and less threatened plants examined. Perhaps it should be further investigated as the susceptibility of island biodiversity hotspots [93] or mountains [94] to climate change might lead to a further increase of the threat status of Greek rare and threatened plants in the country’s key floristic areas, i.e. islands and high mountains.

## Conclusions

We found that a number of extrinsic factors act as drivers of plant vulnerability (viz. habitat, maximal distance), with the most vulnerable taxa being more likely to express particular intrinsic traits (viz. floral complexity, life form).

Our results showed that plants of coastal habitats in Greece are disproportionally assessed as endangered or critically endangered, which is evidently a reflection of human-induced land use change. This result, along with the finding that the vast majority (85.8%) of Greek rare and threatened plants face external threats, mainly of anthropogenic origin [91], point out clearly that human pressure is a main driver of plant vulnerability in the Greek flora, as repeatedly shown elsewhere (e.g.[95]).

On the other hand, although intrinsic plant traits alone (i.e. without taking into account external threats) have been considered insufficient to reliably predict extinction risk [90, 96], these traits can reveal proneness to vulnerability, indicating the most susceptible “victims” when extrinsic drivers are in force. In this respect, our major finding, i.e. that floral complexity is associated with more threatened plants, implies that a higher risk of extinction may occur where insufficient pollination services exist. This is an important finding for applied conservation management.

Declines of wild pollinators are documented worldwide [97, 98] and shown to occur in parallel with declines of plant species [18] as a result of the negative mutual feedbacks to both plants and pollinators. Memmot et al. [99] argue the loss of generalized pollinators is primarily involved in plant decline in northern temperate regions. Species with flowers adapted to long-tongued pollinators have recently been found to be significantly declining in the UK [90]. On the other hand, studies in the tropics and species-rich temperate regions in the Southern Hemisphere highlight the risk of plant extinction caused by the lack of specialized pollinators [15, 19, 100, 101]. These findings make evident that successful plant conservation needs a deeper understanding of plant—pollinator interactions and the causes leading to pollination failure [16, 17]. Based on our results, we suggest that plant—pollinator interactions and floral traits related to floral complexity should be further explored as a mechanism of developing and refining conservation policy and practice. Several conceptual gaps need to be addressed towards this direction. For example, to what extent are plants with complex flowers pollinated by specialized vs. generalized pollinators (e.g. long-tongued), as implied by the association of specialized with generalized species in pollination networks [99, 102]? Or do specialized pollinators actually tend to be rarer than generalized ones as Biesmeijer et al. [18] found for northern Europe? Whatever the answers to these questions are, the assessment of the vulnerability status of pollinator species, especially insects, whose status is still largely unknown [103, 104], and the application of inclusive and holistic plant—pollinator conservation plans are potentially important in long term conservation planning. In this respect, using the Floral Complexity Index for the examination of plant proneness to vulnerability in other regions or biomes (e.g. Mediterranean-type, temperate, tropical) is recommended in order to explore the global validity of this finding for further use in plant conservation programs.

## Supporting Information

S1 Table Dataset. (DOCX)

S1 Text Floral variable level values. (DOCX)

S2 Text The effects of the individual floral complexity components on plant vulnerability. (DOCX)

S3 Text Results of the tests for phylogenetic signal in the Greek rare and threatened plants’ vulnerability as a raw variable (binary trait) and in its residuals in the best fitted GLM according to AIC. (DOCX)
