# Supplementary material for: Lessons from Red Data Books: Plant Vulnerability Increases with Floral Complexity
Source: PLoS One. 2015 Sep 21;10(9):e0138414. doi: 10.1371/journal.pone.0138414 (PMC4577097; doi:10.1371/journal.pone.0138414)
Supplement: S1 Table — (DOCX) [file pone.0138414.s001.docx]

**S1 Table. Dataset.**

| Plant taxon name | Original RDB plant taxon name^1^ | Plant family | Threat status^2^ | Intrinsic variables – FCI^3^ | | | | | | Other intrinsic variables | | | | | | Extrinsic variables | | | | |
| --- | --- | --- | --- | --- | --- | --- | --- | --- | --- | --- | --- | --- | --- | --- | --- | --- | --- | --- | --- | --- |
|  |  |  |  | Floral shape | Floral depth | Floral symmetry | CS^4^ | FRU^5^ | Final FCI | Floral color^6^ | Floral size^7^ | Flowering season | Flowering duration | Life form^8^ | AsR^9^ | Habitat^10^ | Min. altitude (m) | Max. distance (km) | Range-restricted status | PHR^11^ |
| *Acantholimon aegaeum* F. K. Mey. |  | Plumbaginaceae | 0 | 0.83 | 0.4 | 0.2 | 0.4 | 0.15 | 1.98 | W | m | 1 | 2 | WP | no | H | 700 | 103.73 | yes | EAI |
| *Achillea ambrosiaca* (Boiss. & Heldr.) Boiss. |  | Asteraceae | 0 | 0.68 | 0.2 | 0.2 | 0.4 | 0.15 | 1.63 | W | l | 2 | 3 | HP | no | H | 2100 | 0 | yes | GRC |
| *Achillea barbeyana* Heldr. & Heimerl |  | Asteraceae | 0 | 0.68 | 0.2 | 0.2 | 0.4 | 0.15 | 1.63 | W | m | 2 | 2 | HP | no | H | 2350 | 0 | yes | GRC |
| *Achillea occulta* Constantin. & Kalpoutz. |  | Asteraceae | 0 | 0.68 | 0.2 | 0.2 | 0.4 | 0.15 | 1.63 | W | l | 1 | 2 | HP | no | C | 700 | 0 | yes | GRC |
| *Adonis cyllenea* Boiss., Heldr. & Orph. |  | Ranunculaceae | 0 | 0.3 | 0.2 | 0.2 | 0.3 | 0.15 | 1.15 | Ye | l | 1 | 3 | G | yes | G | 1200 | 34.14 | yes | GRC |
| *Aethionema carlsbergii* Strid & Papan. |  | Brassicaceae | 1 | 0.83 | 0.2 | 0.2 | 0.3 | 0.3 | 1.83 | W | s | 2 | 3 | HP | yes | H | 2100 | 5.1 | yes | GRC |
| *Aethionema orbiculatum* (Boiss.) Hayek |  | Brassicaceae | 0 | 0.83 | 0.2 | 0.2 | 0.3 | 0.3 | 1.83 | W | s | 2 | 3 | HP | no | H | 1800 | 0 | yes | GRC |
| *Aethionema retsina* Phitos & Snogerup |  | Brassicaceae | 1 | 0.83 | 0.2 | 0.2 | 0.3 | 0.3 | 1.83 | W | s | 1 | 2 | HP | no | C | 10 | 22.64 | yes | GRC |
| *Ajuga piskoi* Degen & Bald. |  | Lamiaceae | 0 | 1.13 | 0.4 | 0.6 | 0.4 | 0.3 | 2.83 | VPR | l | 2 | 2 | HP | no | W | 392 | 105.48 | yes | GRC |
| *Ajuga pyramidalis* L. |  | Lamiaceae | 0 | 1.13 | 0.6 | 0.6 | 0.4 | 0.3 | 3.03 | B | m | 2 | 3 | G | yes | W | 1400 | 45.82 | no | GRC |
| *Alchemilla aroanica* (Buser) Rothm. |  | Rosaceae | 0 | 0.3 | 0.2 | 0.2 | 0.3 | 0.15 | 1.15 | Ye | s | 2 | 1 | HP | no | A | 1800 | 0 | yes | GRC |
| *Alkanna sartoriana* Boiss. & Heldr. |  | Boraginaceae | 1 | 0.8 | 0.4 | 0.2 | 0.4 | 0.3 | 2.10 | W | s | 1 | 2 | HP | no | M | 30 | 0 | yes | GRC |
| *Allium calamarophilon* Phitos & Tzanoud. |  | Amaryllidaceae | 1 | 0.98 | 0.4 | 0.2 | 0.3 | 0.15 | 2.03 | W | s | 2 | 1 | G | yes | C | 20 | 0 | yes | GRC |
| *Allium chamaemoly* L. |  | Amaryllidaceae | 0 | 0.3 | 0.2 | 0.2 | 0.3 | 0.15 | 1.15 | W | m | 3 | 4 | G | yes | P | 0 | 186.43 | no | GRC |
| *Allium platakisii* Tzanoud. & Kypriot. |  | Amaryllidaceae | 1 | 0.98 | 0.4 | 0.2 | 0.3 | 0.15 | 2.03 | W | s | 2 | 2 | G | yes | C | 0 | 0 | yes | KRI |
| *Allium runemarkii* Trigas & Tzanoud. |  | Amaryllidaceae | 1 | 0.98 | 0.4 | 0.2 | 0.3 | 0.15 | 2.03 | VPR | s | 1 | 1 | G | yes | P | 5 | 0 | yes | GRC |
| *Allium samothracicum* Tzanoud., Strid & Kit Tan |  | Amaryllidaceae | 1 | 1,00 | 0.4 | 0.2 | 0.3 | 0.15 | 2.05 | W | s | 2 | 2 | G | yes | P | 0 | 0 | yes | GRC |
| *Alyssum fragillimum* (Bald.) Rech. f. |  | Brassicaceae | 0 | 0.3 | 0.2 | 0.2 | 0.3 | 0.15 | 1.15 | Ye | s | 2 | 3 | HP | yes | H | 1600 | 6.08 | yes | KRI |
| *Alyssum handelii* Hayek |  | Brassicaceae | 0 | 0.83 | 0.2 | 0.2 | 0.3 | 0.15 | 1.68 | Ye | s | 2 | 3 | HP | no | H | 2500 | 0 | yes | GRC |
| *Alyssum idaeum* Boiss. & Heldr. |  | Brassicaceae | 0 | 0.3 | 0.2 | 0.2 | 0.3 | 0.15 | 1.15 | Ye | s | 1 | 2 | HP | no | H | 1700 | 9.46 | yes | KRI |
| *Alyssum lassiticum* Halácsy |  | Brassicaceae | 0 | 0.83 | 0.4 | 0.2 | 0.3 | 0.15 | 1.88 | Ye | s | 1 | 1 | HP | no | H | 1700 | 141.91 | yes | KRI |
| *Alyssum sphacioticum* Boiss. & Heldr. |  | Brassicaceae | 0 | 0.83 | 0.2 | 0.2 | 0.3 | 0.15 | 1.68 | Ye | s | 1 | 4 | HP | no | H | 1700 | 4.12 | yes | KRI |
| *Alyssum tenium* Halácsy |  | Brassicaceae | 0 | 0.83 | 0.2 | 0.2 | 0.3 | 0.15 | 1.68 | Ye | s | 1 | 2 | WP | no | P | 100 | 0 | yes | GRC |
| *Amelanchier parviflora* subsp. *chelmea* (Halácsy) Ziel. | *Amelanchier chelmea* (Halácsy) Browicz | Rosaceae | 0 | 0.3 | 0.2 | 0.2 | 0.3 | 0.3 | 1.30 | W | m | 1 | 2 | WP | no | W | 1100 | 308.39 | yes | GRC |
| *Anchusa cespitosa* Lam. |  | Boraginaceae | 0 | 0.8 | 0.4 | 0.2 | 0.4 | 0.15 | 1.95 | B | m | 2 | 2 | HP | no | H | 1200 | 19.88 | yes | KRI |
| *Anchusa samothracica* Bigazzi & Selvi |  | Boraginaceae | 1 | 0.8 | 0.4 | 0.2 | 0.4 | 0.15 | 1.95 | B | s | 2 | 2 | HP | no | M | 0 | 0 | yes | GRC |
| *Androcymbium rechingeri* Greuter |  | Colchicaceae | 1 | 0.3 | 0.2 | 0.2 | 0.3 | 0.15 | 1.15 | W | l | 3 | 2 | G | yes | P | 0 | 24.76 | yes | KRI |
| *Anthemis glaberrima* (Rech. f.) Greuter |  | Asteraceae | 1 | 0.68 | 0.2 | 0.2 | 0.4 | 0.15 | 1.63 | W | s | 1 | 2 | T | no | M | 0 | 0 | yes | KRI |
| *Anthemis orbelica* Pančić | *Anthemis macedonica* subsp*. orbelica* (Pančić) Oberpr. &Greuter | Asteraceae | 0 | 0.68 | 0.2 | 0.2 | 0.4 | 0.15 | 1.63 | W | l | 2 | 4 | HP | no | G | 1000 | 23.94 | yes | GRC |
| *Anthemis rhodensis* Boiss. |  | Asteraceae | 0 | 0.68 | 0.2 | 0.2 | 0.4 | 0.15 | 1.63 | W | m | 1 | 2 | HP | no | P | 500 | 17.05 | yes | EAI |
| *Anthemis rosea* Sm. subsp. *rosea* |  | Asteraceae | 0 | 0.68 | 0.2 | 0.2 | 0.4 | 0.15 | 1.63 | VPR | l | 1 | 2 | T | no | G | 1000 | 0 | yes | EAI |
| *Anthemis samariensis* Turland |  | Asteraceae | 0 | 0.68 | 0.4 | 0.2 | 0.4 | 0.15 | 1.83 | W | l | 2 | 2 | HP | no | C | 1675 | 0 | yes | KRI |
| *Anthemis sibthorpii* Griseb. | *Anthemis cretica* subsp. *sibthorpii* (Griseb.) Govaerts | Asteraceae | 1 | 0.68 | 0.2 | 0.2 | 0.4 | 0.15 | 1.63 | Ye | m | 2 | 2 | HP | no | H | 1940 | 0 | yes | GRC |
| *Anthyllis splendens* Willd. |  | Fabaceae | 0 | 1.28 | 0.4 | 0.6 | 0.3 | 0.15 | 2.73 | W | s | 1 | 3 | WP | no | C | 0 | 290.12 | yes | GRC |
| *Aquilegia nigricans* Baumg. |  | Ranunculaceae | 0 | 1,00 | 0.6 | 0.2 | 0.3 | 0.15 | 2.25 | VPR | l | 2 | 2 | HP | yes | W | 1090 | 131.61 | no | GRC |
| *Aquilegia ottonis* Orph. ex Boiss. |  | Ranunculaceae | 0 | 1,00 | 0.6 | 0.2 | 0.3 | 0.15 | 2.25 | B | l | 2 | 4 | HP | no | C | 1100 | 394.73 | yes | GRC |
| *Arabis procurrens* Waldst. & Kit. |  | Brassicaceae | 0 | 0.83 | 0.4 | 0.2 | 0.3 | 0.3 | 2.03 | W | s | 1 | 2 | HP | yes | W | 800 | 26.46 | no | GRC |
| *Arenaria gionae* Gustavsson |  | Caryophyllaceae | 0 | 0.3 | 0.2 | 0.2 | 0.3 | 0.15 | 1.15 | W | m | 2 | 1 | HP | no | C | 1800 | 7.95 | yes | GRC |
| *Arenaria leucadia* Phitos & Strid |  | Caryophyllaceae | 1 | 0.3 | 0.2 | 0.2 | 0.3 | 0.15 | 1.15 | W | s | 1 | 2 | T | no | M | 0 | 215.63 | yes | GRC |
| *Arenaria phitosiana* Greuter & Burdet |  | Caryophyllaceae | 0 | 0.3 | 0.2 | 0.2 | 0.3 | 0.15 | 1.15 | W | s | 1 | 1 | HP | no | M | 5 | 0 | yes | GRC |
| *Armeria johnsenii* Papan. & Kokkini |  | Plumbaginaceae | 0 | 0.68 | 0.2 | 0.2 | 0.4 | 0.15 | 1.63 | W | s | 2 | 3 | HP | no | M | 5 | 0 | yes | GRC |
| *Artemisia inculta* Delile | *Artemisia herba-alba* Asso | Asteraceae | 0 | 0.68 | 0.2 | 0.2 | 0.4 | 0.3 | 1.78 | VPR | s | 3 | 4 | HP | no | P | 0 | 0 | no | KRI |
| *Arum cyrenaicum* Hruby |  | Araceae | 0 | 1.28 | 0.6 | 0.6 | 0.5 | 0.15 | 3.13 | VPR | l | 1 | 2 | G | yes | W | 100 | 12.26 | no | KRI |
| *Arum purpureospathum* P.C. Boyce |  | Araceae | 0 | 1.28 | 0.6 | 0.6 | 0.5 | 0.15 | 3.13 | VPR | l | 1 | 3 | G | yes | R | 0 | 0 | yes | KRI |
| *Asperula baenitzii* Heldr. ex Boiss. |  | Rubiaceae | 1 | 0.8 | 0.2 | 0.2 | 0.4 | 0.15 | 1.75 | W | s | 2 | 4 | G | yes | C | 700 | 47.1 | yes | GRC |
| *Asperula brachyphylla* Trigas & Iatroú |  | Rubiaceae | 0 | 0.8 | 0.4 | 0.2 | 0.4 | 0.3 | 2.10 | W | s | 2 | 2 | HP | no | G | 1100 | 0 | yes | GRC |
| *Asperula crassula* Greuter & Zaffran |  | Rubiaceae | 0 | 0.8 | 0.2 | 0.2 | 0.4 | 0.3 | 1.90 | Ye | s | 1 | 1 | HP | no | P | 20 | 0 | yes | KRI |
| *Asperula elonea* Iatroú & Georgiadis |  | Rubiaceae | 0 | 0.8 | 0.4 | 0.2 | 0.4 | 0.3 | 2.10 | VPR | s | 2 | 2 | HP | no | C | 20 | 38.55 | yes | GRC |
| *Asperula malevonensis* Ehrend. & Schönb.-Tem. |  | Rubiaceae | 0 | 0.8 | 0.4 | 0.2 | 0.4 | 0.3 | 2.10 | VPR | s | 2 | 2 | HP | yes | H | 1600 | 0 | yes | GRC |
| *Asperula muscosa* Boiss. & Heldr. |  | Rubiaceae | 0 | 0.98 | 0.2 | 0.2 | 0.4 | 0.3 | 2.08 | W | s | 2 | 4 | G | yes | W | 1200 | 0 | yes | GRC |
| *Asperula naufraga* Ehrend. & Gutermann |  | Rubiaceae | 1 | 0.8 | 0.2 | 0.2 | 0.4 | 0.3 | 1.90 | VPR | s | 2 | 2 | HP | no | C | 15 | 20.23 | yes | GRC |
| *Asperula saxicola* Ehrend*.* |  | Rubiaceae | 0 | 0.8 | 0.2 | 0.2 | 0.4 | 0.3 | 1.90 | Ye | s | 2 | 2 | HP | no | C | 550 | 20.21 | yes | GRC |
| *Astragalus agraniotii* Orph. ex Boiss. |  | Fabaceae | 1 | 1.28 | 0.4 | 0.6 | 0.4 | 0.3 | 2.98 | VPR | m | 2 | 1 | HP | no | H | 1700 | 0 | yes | GRC |
| *Astragalus drupaceus* Orph. ex Boiss. |  | Fabaceae | 0 | 1.28 | 0.6 | 0.6 | 0.4 | 0.3 | 3.18 | Ye | l | 1 | 2 | HP | no | P | 100 | 113.68 | yes | GRC |
| *Astragalus idaeus* Bunge |  | Fabaceae | 1 | 1.28 | 0.4 | 0.6 | 0.4 | 0.3 | 2.98 | Ye | m | 1 | 1 | HP | no | H | 1800 | 61.61 | yes | KRI |
| *Astragalus laconicus* Iatroú & Kit Tan |  | Fabaceae | 0 | 1.28 | 0.6 | 0.6 | 0.4 | 0.3 | 3.18 | VPR | m | 1 | 2 | HP | no | P | 100 | 43.93 | yes | GRC |
| *Astragalus maniaticus* Kit Tan & Strid |  | Fabaceae | 0 | 1.28 | 0.6 | 0.6 | 0.4 | 0.3 | 3.18 | VPR | m | 1 | 2 | HP | no | R | 0 | 142.83 | yes | GRC |
| *Asyneuma giganteum* (Boiss.) Bornm. |  | Campanulaceae | 0 | 0.3 | 0.2 | 0.2 | 0.4 | 0.3 | 1.40 | VPR | m | 2 | 3 | HP | no | C | 200 | 120.62 | yes | EAI |
| *Aubrieta erubescens* Griseb. |  | Brassicaceae | 0 | 0.83 | 0.4 | 0.2 | 0.3 | 0.15 | 1.88 | W | m | 1 | 3 | HP | no | H | 800 | 0 | yes | GRC |
| *Aubrieta glabrescens* Turrill |  | Brassicaceae | 0 | 0.83 | 0.4 | 0.2 | 0.3 | 0.15 | 1.88 | VPR | m | 2 | 2 | HP | no | H | 2370 | 0 | yes | GRC |
| *Aubrieta scyria* Halácsy |  | Brassicaceae | 0 | 0.83 | 0.4 | 0.2 | 0.3 | 0.15 | 1.88 | VPR | l | 1 | 1 | HP | no | C | 200 | 44.47 | yes | GRC |
| *Bellevalia brevipedicellata* Turrill |  | Asparagaceae | 1 | 1,00 | 0.4 | 0.2 | 0.4 | 0.3 | 2.30 | W | s | 1 | 3 | G | yes | P | 0 | 70.84 | yes | KRI |
| *Bellevalia edirnensis* Özhatay & B. Mathew |  | Asparagaceae | 1 | 0.98 | 0.6 | 0.2 | 0.4 | 0.3 | 2.48 | W | m | 1 | 1 | G | yes | R | 70 | 0 | yes | GRC |
| *Bellevalia sitiaca* Kypriot. & Tzanoud. |  | Asparagaceae | 0 | 0.98 | 0.4 | 0.2 | 0.4 | 0.3 | 2.28 | W | s | 1 | 2 | G | yes | P | 0 | 66.18 | yes | KRI |
| *Beta nana* Boiss. & Heldr. |  | Amaranthaceae | 0 | 0.3 | 0.2 | 0.2 | 0.4 | 0.3 | 1.40 | Gr | s | 2 | 3 | HP | no | H | 1900 | 343.14 | yes | GRC |
| *Biarum davisii* Turrill |  | Araceae | 0 | 1.28 | 0.6 | 0.6 | 0.5 | 0.15 | 3.13 | Ye | l | 3 | 2 | G | yes | P | 0 | 236.21 | yes | KRI |
| *Biarum fraasianum* (Schott) N.E. Br. |  | Araceae | 1 | 1.28 | 0.6 | 0.6 | 0.5 | 0.15 | 3.13 | VPR | l | 1 | 2 | G | yes | P | 100 | 0 | yes | GRC |
| *Biebersteinia orphanidis* Boiss. |  | Biebersteiniaceae | 1 | 0.3 | 0.4 | 0.2 | 0.3 | 0.3 | 1.50 | VPR | s | 1 | 2 | G | yes | G | 1350 | 20.38 | no | GRC |
| *Bolanthus creutzburgii* Greuter |  | Caryophyllaceae | 0 | 0.83 | 0.2 | 0.2 | 0.3 | 0.15 | 1.68 | W | s | 2 | 3 | HP | no | P | 1550 | 104.51 | yes | KRI |
| *Bonannia graeca* (L.) Halácsy |  | Apiaceae | 1 | 0.3 | 0.2 | 0.2 | 0.3 | 0.15 | 1.15 | Ye | l | 1 | 2 | HP | no | R | 160 | 262.32 | no | GRC |
| *Bongardia chrysogonum* (L.) Spach |  | Berberidaceae | 1 | 0.3 | 0.2 | 0.2 | 0.3 | 0.3 | 1.30 | Ye | l | 1 | 2 | G | yes | R | 350 | 343.95 | no | EAI |
| *Brassica nivalis* Boiss. & Heldr. subsp. *nivalis* | *Coincya nivalis* (Boiss. & Heldr.) Greuter & Burdet | Brassicaceae | 0 | 0.83 | 0.4 | 0.2 | 0.3 | 0.3 | 2.03 | Ye | m | 2 | 3 | HP | no | H | 1900 | 0 | yes | GRC |
| *Bubon arachnoideum* (Boiss. & Orph.) Hand | *Athamanta arachnoidea* Boiss. & Orph. | Apiaceae | 0 | 0.3 | 0.2 | 0.2 | 0.3 | 0.15 | 1.15 | W | l | 2 | 2 | HP | no | C | 450 | 62.07 | yes | GRC |
| *Bupleurum aira* Snogerup |  | Apiaceae | 0 | 0.3 | 0.2 | 0.2 | 0.3 | 0.15 | 1.15 | Ye | s | 1 | 3 | T | no | P | 100 | 6.72 | yes | GRC |
| *Bupleurum capillare* Boiss. & Heldr. |  | Apiaceae | 0 | 0.3 | 0.2 | 0.2 | 0.3 | 0.15 | 1.15 | Ye | s | 2 | 3 | T | no | R | 500 | 32.94 | yes | GRC |
| *Bupleurum gaudianum* Snogerup |  | Apiaceae | 0 | 0.3 | 0.2 | 0.2 | 0.3 | 0.15 | 1.15 | Ye | s | 1 | 2 | T | no | P | 0 | 0 | yes | KRI |
| *Bupleurum greuteri* Snogerup |  | Apiaceae | 0 | 0.3 | 0.2 | 0.2 | 0.3 | 0.15 | 1.15 | Ye | s | 1 | 2 | T | no | P | 0 | 76.24 | yes | GRC |
| *Bupleurum kakiskalae* Greuter |  | Apiaceae | 1 | 0.3 | 0.2 | 0.2 | 0.3 | 0.15 | 1.15 | Ye | s | 2 | 3 | HP | no | C | 1450 | 0 | yes | KRI |
| *Calamintha cretica* (L.) Lam. | *Satureja cretica* (L.) Briq. | Lamiaceae | 0 | 1.13 | 0.4 | 0.6 | 0.4 | 0.3 | 2.83 | W | s | 1 | 1 | HP | no | P | 100 | 20.18 | yes | KRI |
| *Campanula aizoides* Zaffran ex Greuter |  | Campanulaceae | 0 | 0.8 | 0.6 | 0.2 | 0.4 | 0.3 | 2.30 | B | m | 2 | 3 | HP | no | H | 1250 | 353.45 | yes | KRI |
| *Campanula aizoon* Boiss. & Spruner |  | Campanulaceae | 0 | 0.8 | 0.6 | 0.2 | 0.4 | 0.3 | 2.30 | B | m | 2 | 3 | HP | no | H | 1600 | 30.91 | yes | GRC |
| *Campanula asperuloides* (Boiss. & Orph.) Engl. |  | Campanulaceae | 0 | 0.83 | 0.4 | 0.2 | 0.4 | 0.15 | 1.98 | B | s | 2 | 3 | HP | no | C | 400 | 133.45 | yes | GRC |
| *Campanula columnaris* Contandr., Quézel & Zaffran |  | Campanulaceae | 0 | 0.8 | 0.6 | 0.2 | 0.4 | 0.3 | 2.30 | B | m | 2 | 3 | HP | no | H | 1600 | 4.5 | yes | GRC |
| *Campanula cymaea* Phitos |  | Campanulaceae | 0 | 0.8 | 0.6 | 0.2 | 0.4 | 0.3 | 2.30 | VPR | l | 1 | 2 | HP | no | C | 10 | 6.52 | yes | GRC |
| *Campanula cymbalaria* Sm. |  | Campanulaceae | 0 | 0.8 | 0.4 | 0.2 | 0.4 | 0.15 | 1.95 | B | m | 2 | 1 | HP | no | C | 1250 | 0 | no | EAI |
| *Campanula garganica* subsp. *acarnanica* (Damboldt) Damboldt |  | Campanulaceae | 0 | 0.8 | 0.2 | 0.2 | 0.4 | 0.15 | 1.75 | B | m | 1 | 3 | HP | yes | C | 800 | 6.41 | yes | GRC |
| *Campanula garganica* subsp*. cephallenica* (Feer) Hayek |  | Campanulaceae | 0 | 0.3 | 0.2 | 0.2 | 0.4 | 0.3 | 1.40 | B | m | 1 | 3 | HP | no | C | 50 | 99.71 | yes | GRC |
| *Campanula hierapetrae* Rech. f. |  | Campanulaceae | 0 | 0.8 | 0.4 | 0.2 | 0.4 | 0.15 | 1.95 | B | m | 2 | 2 | G | yes | C | 1000 | 0 | yes | KRI |
| *Campanula incurva* A. DC. |  | Campanulaceae | 0 | 0.8 | 0.6 | 0.2 | 0.4 | 0.15 | 2.15 | B | l | 2 | 2 | HP | no | C | 20 | 405.64 | yes | GRC |
| *Campanula laciniata* L. |  | Campanulaceae | 0 | 0.8 | 0.4 | 0.2 | 0.4 | 0.3 | 2.10 | B | l | 1 | 3 | HP | no | C | 0 | 300.23 | yes | KRI |
| *Campanula merxmuelleri* Phitos |  | Campanulaceae | 0 | 0.8 | 0.6 | 0.2 | 0.4 | 0.15 | 2.15 | VPR | m | 1 | 2 | HP | no | C | 30 | 86.5 | yes | GRC |
| *Campanula pangea* Hartvig |  | Campanulaceae | 0 | 0.8 | 0.6 | 0.2 | 0.4 | 0.3 | 2.30 | VPR | l | 2 | 2 | HP | yes | G | 1000 | 0 | yes | GRC |
| *Campanula papillosa* Halácsy |  | Campanulaceae | 1 | 0.8 | 0.6 | 0.2 | 0.4 | 0.15 | 2.15 | VPR | m | 2 | 3 | HP | yes | H | 1900 | 0 | yes | GRC |
| *Campanula reiseri* Halácsy |  | Campanulaceae | 0 | 0.8 | 0.6 | 0.2 | 0.4 | 0.15 | 2.15 | VPR | l | 1 | 2 | HP | no | C | 10 | 291.15 | yes | GRC |
| *Campanula rupestris* Sm. |  | Campanulaceae | 0 | 0.8 | 0.6 | 0.2 | 0.4 | 0.3 | 2.30 | VPR | m | 1 | 2 | HP | no | C | 160 | 0 | yes | GRC |
| *Campanula samothracica* (Degen) Greuter & Burdet subsp*. samothracica* |  | Campanulaceae | 0 | 1,00 | 0.6 | 0.2 | 0.4 | 0.15 | 2.35 | VPR | l | 2 | 2 | HP | no | C | 50 | 0 | yes | GRC |
| *Campanula sartorii* Boiss. & Heldr. |  | Campanulaceae | 0 | 0.8 | 0.4 | 0.2 | 0.4 | 0.3 | 2.10 | W | s | 1 | 2 | HP | no | C | 100 | 40.42 | yes | GRC |
| *Campanula saxatilis* L. subsp. *saxatilis* |  | Campanulaceae | 0 | 0.98 | 0.6 | 0.2 | 0.4 | 0.15 | 2.33 | B | m | 1 | 3 | HP | no | C | 0 | 70.99 | yes | KRI |
| *Campanula wanneri* Rochel | *Symphyandra wanneri* (Rochel) Heuff. | Campanulaceae | 0 | 1,00 | 0.6 | 0.2 | 0.5 | 0.3 | 2.60 | VPR | l | 2 | 1 | HP | no | C | 1500 | 10.13 | no | GRC |
| *Carlina diae* (Rech. f.) Meusel & Kastner |  | Asteraceae | 1 | 0.68 | 0.6 | 0.2 | 0.4 | 0.15 | 2.03 | Ye | l | 2 | 2 | HP | no | C | 0 | 95.03 | yes | KRI |
| *Centaurea achaia* subsp. *corinthiaca* (Boiss. & Heldr.) Phitos & T. Georgiadis |  | Asteraceae | 0 | 0.68 | 0.6 | 0.2 | 0.4 | 0.15 | 2.03 | W | l | 2 | 3 | HP | no | G | 50 | 16.23 | yes | GRC |
| *Centaurea aetolica* Phitos & T. Georgiadis |  | Asteraceae | 1 | 0.68 | 0.6 | 0.2 | 0.4 | 0.15 | 2.03 | VPR | l | 1 | 2 | HP | no | R | 2 | 36.92 | yes | GRC |
| *Centaurea alba* subsp. *subciliaris* (Boiss. & Heldr.) Dostál | *Centaurea subciliaris* subsp. *acarnanica* Matthäs | Asteraceae | 0 | 0.68 | 0.6 | 0.2 | 0.4 | 0.15 | 2.03 | VPR | l | 2 | 2 | HP | no | G | 700 | 129.63 | yes | GRC |
| *Centaurea argentea* L. subsp*. argentea* |  | Asteraceae | 0 | 0.68 | 0.6 | 0.2 | 0.4 | 0.15 | 2.03 | Ye | m | 1 | 2 | HP | no | C | 0 | 109.6 | yes | KRI |
| *Centaurea argentea* subsp*. chionantha* (Turland & L. Chilton) Greuter |  | Asteraceae | 0 | 0.68 | 0.6 | 0.2 | 0.4 | 0.15 | 2.03 | W | m | 1 | 2 | HP | no | C | 0 | 22.17 | yes | KRI |
| *Centaurea athoa* DC. subsp. *athoa* |  | Asteraceae | 0 | 0.68 | 0.6 | 0.2 | 0.4 | 0.15 | 2.03 | Ye | l | 2 | 2 | HP | no | H | 1400 | 0 | yes | GRC |
| *Centaurea attica* subsp. *megarensis* (Halácsy & Hayek) Dostál |  | Asteraceae | 0 | 0.68 | 0.6 | 0.2 | 0.4 | 0.15 | 2.03 | W | l | 1 | 2 | HP | no | G | 550 | 0 | yes | GRC |
| *Centaurea baldaccii* Degen ex Bald. | *Cyanus baldaccii* (Bald.) Holub | Asteraceae | 0 | 0.68 | 0.6 | 0.2 | 0.4 | 0.15 | 2.03 | W | l | 2 | 3 | G | yes | H | 1800 | 0 | yes | KRI |
| *Centaurea carystea* Trigas & Constantin*.* |  | Asteraceae | 1 | 0.68 | 0.6 | 0.2 | 0.4 | 0.15 | 2.03 | Ye | l | 2 | 2 | HP | no | G | 1200 | 0 | yes | GRC |
| *Centaurea charrelii* Halácsy & Dörfl. |  | Asteraceae | 1 | 0.68 | 0.6 | 0.2 | 0.4 | 0.15 | 2.03 | Ye | l | 2 | 2 | HP | no | W | 500 | 281.32 | yes | GRC |
| *Centaurea chrysocephala* Phitos & T. Georgiadis |  | Asteraceae | 0 | 0.68 | 0.6 | 0.2 | 0.4 | 0.15 | 2.03 | W | m | 1 | 2 | HP | no | C | 350 | 64.87 | yes | GRC |
| *Centaurea cithaeronea* Phitos & Constantin. |  | Asteraceae | 1 | 0.68 | 0.6 | 0.2 | 0.4 | 0.15 | 2.03 | Ye | m | 2 | 2 | HP | no | G | 1000 | 43.48 | yes | GRC |
| *Centaurea heldreichii* Halácsy |  | Asteraceae | 0 | 0.68 | 0.6 | 0.2 | 0.4 | 0.15 | 2.03 | VPR | l | 1 | 2 | HP | no | C | 3 | 0 | yes | GRC |
| *Centaurea incompleta* Halácsy |  | Asteraceae | 1 | 0.68 | 0.6 | 0.2 | 0.4 | 0.15 | 2.03 | Ye | l | 2 | 1 | HP | no | C | 400 | 0 | yes | GRC |
| *Centaurea kalambakensis* Freyn & Sint. |  | Asteraceae | 0 | 0.68 | 0.6 | 0.2 | 0.4 | 0.15 | 2.03 | VPR | m | 1 | 2 | HP | no | C | 250 | 0 | yes | GRC |
| *Centaurea lactucifolia* Boiss. |  | Asteraceae | 0 | 0.68 | 0.6 | 0.2 | 0.4 | 0.15 | 2.03 | Ye | l | 1 | 2 | HP | no | C | 50 | 54.41 | yes | EAI |
| *Centaurea lancifolia* Spreng. |  | Asteraceae | 1 | 0.68 | 0.6 | 0.2 | 0.4 | 0.15 | 2.03 | Ye | l | 2 | 3 | HP | no | C | 1700 | 134.33 | yes | KRI |
| *Centaurea leonidia* Kalpoutz. & Constantin. |  | Asteraceae | 1 | 0.68 | 0.6 | 0.2 | 0.4 | 0.15 | 2.03 | VPR | l | 1 | 2 | HP | no | C | 450 | 0 | yes | GRC |
| *Centaurea litochorea* T. Georgiadis & Phitos |  | Asteraceae | 0 | 0.68 | 0.6 | 0.2 | 0.4 | 0.15 | 2.03 | Ye | l | 2 | 2 | HP | no | C | 830 | 10.83 | yes | GRC |
| *Centaurea messenicolasiana* T. Georgiadis, Dimitrellos & Routsi |  | Asteraceae | 0 | 0.68 | 0.6 | 0.2 | 0.4 | 0.15 | 2.03 | W | l | 2 | 3 | HP | no | R | 500 | 0 | yes | GRC |
| *Centaurea musarum* Boiss. & Orph. |  | Asteraceae | 1 | 0.68 | 0.6 | 0.2 | 0.4 | 0.15 | 2.03 | Ye | l | 2 | 2 | HP | no | C | 1500 | 0 | yes | GRC |
| *Centaurea niederi* Heldr. |  | Asteraceae | 0 | 0.68 | 0.6 | 0.2 | 0.4 | 0.15 | 2.03 | VPR | l | 1 | 2 | HP | no | C | 0 | 41.16 | yes | GRC |
| *Centaurea paxorum* Phitos & T. Georgiadis |  | Asteraceae | 0 | 0.68 | 0.6 | 0.2 | 0.4 | 0.15 | 2.03 | VPR | l | 1 | 3 | HP | no | M | 0 | 0 | yes | GRC |
| *Centaurea peucedanifolia* Boiss. & Orph*.* |  | Asteraceae | 0 | 0.68 | 0.6 | 0.2 | 0.4 | 0.15 | 2.03 | VPR | l | 1 | 3 | HP | no | C | 20 | 0 | yes | GRC |
| *Centaurea poculatoris* Greuter |  | Asteraceae | 0 | 0.68 | 0.6 | 0.2 | 0.4 | 0.15 | 2.03 | VPR | m | 1 | 2 | HP | no | C | 50 | 8.38 | yes | KRI |
| *Centaurea princeps* Boiss. & Heldr. |  | Asteraceae | 0 | 0.68 | 0.6 | 0.2 | 0.4 | 0.15 | 2.03 | W | l | 2 | 2 | HP | no | C | 1200 | 0 | yes | GRC |
| *Centaurea pseudocadmea* Wagenitz |  | Asteraceae | 0 | 0.68 | 0.6 | 0.2 | 0.4 | 0.15 | 2.03 | VPR | l | 1 | 2 | HP | yes | G | 800 | 148.4 | yes | GRC |
| *Centaurea pumilio* L. |  | Asteraceae | 0 | 0.68 | 0.6 | 0.2 | 0.4 | 0.15 | 2.03 | VPR | l | 1 | 2 | HP | yes | M | 0 | 416.23 | no | GRC |
| *Centaurea rechingeri* Phitos |  | Asteraceae | 0 | 0.68 | 0.6 | 0.2 | 0.4 | 0.15 | 2.03 | VPR | l | 1 | 2 | HP | no | C | 0 | 297.84 | yes | GRC |
| *Centaurea tuntasia* Halácsy |  | Asteraceae | 1 | 0.68 | 0.6 | 0.2 | 0.4 | 0.15 | 2.03 | W | l | 2 | 2 | HP | no | R | 99 | 22.82 | yes | GRC |
| *Centaurea vlachorum* Hartvig |  | Asteraceae | 0 | 0.68 | 0.6 | 0.2 | 0.4 | 0.15 | 2.03 | VPR | l | 2 | 2 | HP | yes | H | 1700 | 4.86 | yes | GRC |
| *Centaurea xylobasis* Rech. f. |  | Asteraceae | 0 | 0.68 | 0.6 | 0.2 | 0.4 | 0.15 | 2.03 | Ye | m | 2 | 2 | HP | no | C | 1200 | 0 | yes | EAI |
| *Centranthus sieberi* Heldr. |  | Caprifoliaceae | 0 | 0.83 | 0.6 | 0.2 | 0.4 | 0.3 | 2.33 | VPR | l | 2 | 3 | HP | no | H | 1800 | 0 | yes | KRI |
| *Cephalanthera cucullata* Boiss. & Heldr. |  | Orchidaceae | 1 | 1.35 | 0.2 | 0.6 | 0.4 | 0.3 | 2.85 | W | m | 1 | 2 | G | yes | W | 500 | 151.76 | yes | KRI |
| *Cephalanthera epipactoides* Fisch. & C.A. Mey. |  | Orchidaceae | 0 | 1.35 | 0.2 | 0.6 | 0.4 | 0.3 | 2.85 | W | m | 1 | 3 | G | yes | W | 200 | 549.86 | no | EAI |
| *Cephalaria squamiflora* (Sieber) Greuter |  | Caprifoliaceae | 0 | 0.68 | 0.4 | 0.2 | 0.4 | 0.15 | 1.83 | W | l | 2 | 2 | HP | no | C | 460 | 497.08 | yes | KRI |
| *Cephalaria tenuiloba* Strid |  | Caprifoliaceae | 0 | 0.68 | 0.4 | 0.2 | 0.4 | 0.15 | 1.83 | Ye | m | 2 | 2 | HP | no | H | 1500 | 0 | yes | GRC |
| *Cerastium dominici* Kit Tan & R.R. Mill |  | Caryophyllaceae | 0 | 0.8 | 0.2 | 0.2 | 0.3 | 0.15 | 1.65 | W | s | 1 | 2 | T | no | P | 250 | 0 | yes | EAI |
| *Cerastium illyricum* subsp. *crinitum* (Lonsing) P.D. Sell & Whitehead |  | Caryophyllaceae | 0 | 0.8 | 0.2 | 0.2 | 0.3 | 0.15 | 1.65 | W | m | 1 | 2 | T | no | G | 500 | 4.28 | yes | GRC |
| *Cerastium runemarkii* Möschl & Rech. f. |  | Caryophyllaceae | 0 | 0.3 | 0.2 | 0.2 | 0.3 | 0.15 | 1.15 | W | m | 1 | 2 | HP | no | C | 950 | 138.06 | yes | GRC |
| *Cerastium theophrasti* Merxm. & Strid |  | Caryophyllaceae | 0 | 0.3 | 0.2 | 0.2 | 0.3 | 0.15 | 1.15 | W | m | 2 | 3 | HP | no | H | 2500 | 0 | yes | GRC |
| *Chaerophyllum creticum* Boiss. & Heldr. |  | Apiaceae | 0 | 0.3 | 0.2 | 0.2 | 0.3 | 0.15 | 1.15 | Ye | l | 2 | 4 | HP | yes | R | 1050 | 93.34 | yes | KRI |
| *Cicer graecum* Orph. ex Boiss. |  | Fabaceae | 1 | 1.28 | 0.4 | 0.6 | 0.3 | 0.15 | 2.73 | VPR | l | 1 | 2 | HP | no | W | 800 | 16.92 | yes | GRC |
| *Cicuta virosa* L. |  | Apiaceae | 0 | 0.3 | 0.2 | 0.2 | 0.3 | 0.15 | 1.15 | W | l | 2 | 2 | HP | yes | A | 470 | 57.88 | no | GRC |
| *Cirsium steirolepis* Petr. |  | Asteraceae | 0 | 0.68 | 0.6 | 0.2 | 0.4 | 0.15 | 2.03 | VPR | l | 2 | 3 | HP | no | R | 300 | 10.25 | yes | EAI |
| *Cistanche phelypaea* (L.) Cout. |  | Orobanchaceae | 0 | 1.13 | 0.6 | 0.6 | 0.4 | 0.3 | 3.03 | Ye | l | 1 | 2 | G | yes | M | 0 | 127.63 | no | KRI |
| *Clematis elisabethae-carolae* Greuter |  | Ranunculaceae | 1 | 0.3 | 0.2 | 0.2 | 0.3 | 0.3 | 1.30 | W | m | 2 | 2 | HP | no | H | 1250 | 11.63 | yes | KRI |
| *Clinopodium taygeteum* (P.H. Davis) Brauchler & Heubl | *Micromeria taygetea* P. H. Davis | Lamiaceae | 1 | 1.13 | 0.4 | 0.6 | 0.4 | 0.3 | 2.83 | VPR | s | 2 | 4 | HP | no | C | 1600 | 0 | yes | GRC |
| *Colchicum asteranthum* Vassil. & K.M. Perss. |  | Colchicaceae | 0 | 0.8 | 0.2 | 0.2 | 0.4 | 0.15 | 1.75 | W | l | 3 | 2 | G | yes | G | 950 | 0 | yes | GRC |
| *Colchicum burttii* Meikle |  | Colchicaceae | 0 | 0.8 | 0.2 | 0.2 | 0.4 | 0.15 | 1.75 | VPR | l | 3 | 2 | G | yes | P | 0 | 23.67 | no | EAI |
| *Colchicum chimonanthum* K.M. Perss*.* |  | Colchicaceae | 0 | 0.8 | 0.2 | 0.2 | 0.4 | 0.15 | 1.75 | W | l | 3 | 2 | G | yes | G | 100 | 105.77 | yes | GRC |
| *Colchicum euboeum* (Boiss.) K.M. Perss. |  | Colchicaceae | 0 | 0.8 | 0.6 | 0.2 | 0.4 | 0.15 | 2.15 | VPR | l | 2 | 2 | G | yes | G | 900 | 41.39 | yes | GRC |
| *Colchicum lingulatum* Boiss. & Spruner subsp. *lingulatum* |  | Colchicaceae | 0 | 0.8 | 0.6 | 0.2 | 0.4 | 0.15 | 2.15 | VPR | l | 2 | 2 | G | yes | P | 200 | 116.26 | yes | GRC |
| *Colchicum parnassicum* Sartori, Orph. & Heldr. ex Boiss. |  | Colchicaceae | 0 | 0.8 | 0.6 | 0.2 | 0.4 | 0.15 | 2.15 | VPR | l | 2 | 2 | G | yes | H | 1800 | 68.2 | yes | GRC |
| *Colchicum peloponnesiacum* Rech. f. & P.H. Davis |  | Colchicaceae | 0 | 0.8 | 0.2 | 0.2 | 0.4 | 0.15 | 1.75 | VPR | l | 3 | 2 | G | yes | P | 800 | 114.94 | yes | GRC |
| *Colchicum pulchellum* K.M. Perss. |  | Colchicaceae | 0 | 0.8 | 0.6 | 0.2 | 0.4 | 0.15 | 2.15 | VPR | l | 2 | 2 | G | yes | H | 1400 | 104.89 | yes | GRC |
| *Colchicum rausii* K.M. Perss. |  | Colchicaceae | 0 | 0.8 | 0.6 | 0.2 | 0.4 | 0.15 | 2.15 | VPR | l | 2 | 1 | G | yes | H | 1500 | 127.15 | yes | GRC |
| *Colchicum soboliferum* (Fisch. & C A. Mey.) Stef. |  | Colchicaceae | 0 | 0.8 | 0.2 | 0.2 | 0.3 | 0.15 | 1.65 | W | l | 1 | 4 | G | yes | G | 0 | 447.05 | no | GRC |
| *Colchicum stevenii* Kunth |  | Colchicaceae | 0 | 0.8 | 0.2 | 0.2 | 0.4 | 0.15 | 1.75 | VPR | l | 3 | 2 | G | yes | P | 0 | 5.23 | no | EAI |
| *Colchicum zahnii* Heldr. | *Colchicum psaridis* Heldr. ex Halácsy | Colchicaceae | 0 | 0.8 | 0.2 | 0.2 | 0.4 | 0.15 | 1.75 | VPR | l | 3 | 3 | G | yes | P | 0 | 120.56 | yes | GRC |
| *Consolida arenaria* Carlström |  | Ranunculaceae | 1 | 0.98 | 0.4 | 0.6 | 0.4 | 0.15 | 2.53 | VPR | m | 1 | 2 | T | no | M | 0 | 479.27 | yes | EAI |
| *Consolida brevicornis* (Vis.) Soó |  | Ranunculaceae | 0 | 0.98 | 0.4 | 0.6 | 0.4 | 0.3 | 2.68 | VPR | m | 1 | 3 | T | no | P | 0 | 88.87 | no | GRC |
| *Consolida samia* P.H. Davis |  | Ranunculaceae | 1 | 0.98 | 0.6 | 0.6 | 0.4 | 0.15 | 2.73 | VPR | l | 1 | 2 | T | no | P | 800 | 0 | yes | EAI |
| *Consolida tenuissima* (Sm.) Soó |  | Ranunculaceae | 0 | 0.98 | 0.4 | 0.6 | 0.4 | 0.3 | 2.68 | B | m | 1 | 3 | T | no | P | 250 | 110.18 | yes | GRC |
| *Consolida tuntasiana* (Halácsy) Soó |  | Ranunculaceae | 1 | 0.98 | 0.2 | 0.6 | 0.4 | 0.15 | 2.33 | VPR | m | 1 | 2 | T | no | G | 750 | 144.61 | yes | GRC |
| *Convolvulus argyrothamnos* Greuter |  | Convolvulaceae | 1 | 0.3 | 0.2 | 0.2 | 0.4 | 0.15 | 1.25 | W | l | 2 | 2 | HP | no | C | 150 | 0 | yes | KRI |
| *Convolvulus mairei* Halácsy |  | Convolvulaceae | 0 | 0.8 | 0.4 | 0.2 | 0.5 | 0.15 | 2.05 | W | m | 2 | 3 | G | yes | A | 700 | 99.59 | yes | GRC |
| *Corydalis thasia* (Stoj. & Kitan.) Stoj. & Kitan*.* |  | Papaveraceae | 0 | 1.28 | 0.6 | 0.6 | 0.3 | 0.3 | 3.08 | VPR | m | 1 | 2 | G | yes | W | 450 | 319.21 | yes | GRC |
| *Cotoneaster parnassicus* Boiss. & Heldr. |  | Rosaceae | 0 | 0.3 | 0.2 | 0.2 | 0.3 | 0.15 | 1.15 | VPR | m | 2 | 3 | WP | no | W | 1100 | 359.13 | yes | GRC |
| *Crepis arcuata* Kamari & Strid |  | Asteraceae | 1 | 0.68 | 0.4 | 0.2 | 0.4 | 0.15 | 1.83 | Ye | l | 2 | 2 | HP | no | H | 2400 | 0 | yes | GRC |
| *Crepis athoa* Boiss. |  | Asteraceae | 0 | 0.68 | 0.4 | 0.2 | 0.4 | 0.15 | 1.83 | Ye | l | 2 | 2 | HP | no | H | 1500 | 0 | yes | GRC |
| *Crepis auriculifolia* Spreng. |  | Asteraceae | 0 | 0.68 | 0.4 | 0.2 | 0.4 | 0.15 | 1.83 | Ye | l | 2 | 3 | HP | no | C | 500 | 181.58 | yes | KRI |
| *Crepis heldreichiana* (Kuntze) Greuter |  | Asteraceae | 0 | 0.68 | 0.4 | 0.2 | 0.4 | 0.15 | 1.83 | Ye | m | 2 | 4 | HP | no | H | 1450 | 38.1 | yes | GRC |
| *Crepis merxmuelleri* Kamari & Hartvig |  | Asteraceae | 0 | 0.68 | 0.4 | 0.2 | 0.4 | 0.15 | 1.83 | Ye | m | 2 | 2 | HP | no | G | 1000 | 9.91 | yes | GRC |
| *Crepis sibthorpiana* Boiss. & Heldr. |  | Asteraceae | 0 | 0.68 | 0.4 | 0.2 | 0.4 | 0.15 | 1.83 | Ye | l | 2 | 4 | HP | no | H | 1500 | 84.04 | yes | KRI |
| *Crocus biflorus* subsp. *stridii* (Papan. & Zacharof) B. Mathew |  | Iridaceae | 0 | 0.8 | 0.2 | 0.2 | 0.4 | 0.15 | 1.75 | W | l | 3 | 2 | G | yes | G | 30 | 160.21 | yes | GRC |
| *Crocus goulimyi* Turrill |  | Iridaceae | 0 | 0.8 | 0.2 | 0.2 | 0.4 | 0.15 | 1.75 | VPR | l | 3 | 2 | G | yes | P | 300 | 43.57 | yes | GRC |
| *Crocus pelistericus* Pulević |  | Iridaceae | 0 | 0.8 | 0.6 | 0.2 | 0.4 | 0.15 | 2.15 | VPR | l | 1 | 2 | G | yes | A | 1900 | 41.96 | yes | GRC |
| *Crocus robertianus* CD. Brickell |  | Iridaceae | 0 | 0.8 | 0.2 | 0.2 | 0.4 | 0.15 | 1.75 | W | l | 3 | 2 | G | yes | W | 150 | 278.61 | yes | GRC |
| *Crocus speciosus* M. Bieb. subsp*. speciosus* |  | Iridaceae | 0 | 0.8 | 0.2 | 0.2 | 0.4 | 0.15 | 1.75 | VPR | l | 3 | 3 | G | yes | R | 800 | 186.88 | no | GRC |
| *Cruciata taurica* subsp*. euboea* (Ehrend.) Ehrend. |  | Rubiaceae | 0 | 0.3 | 0.2 | 0.2 | 0.4 | 0.3 | 1.40 | Ye | s | 1 | 2 | HP | no | H | 1000 | 193.6 | yes | GRC |
| *Cuscuta atrans* Feinbrun |  | Convolvulaceae | 0 | 0.68 | 0.2 | 0.2 | 0.4 | 0.15 | 1.63 | W | s | 2 | 2 | T | no | H | 1400 | 132.76 | yes | KRI |
| *Cyathophylla chlorifolia* (Poir.) Bocq. & Strid |  | Caryophyllaceae | 0 | 0.83 | 0.4 | 0.2 | 0.3 | 0.15 | 1.88 | VPR | m | 1 | 2 | T | no | H | 800 | 385.59 | no | EAI |
| *Cyclamen persicum* Mill. |  | Primulaceae | 0 | 1,00 | 0.4 | 0.2 | 0.4 | 0.15 | 2.15 | W | l | 1 | 5 | G | yes | W | 0 | 363.7 | no | EAI |
| *Cynara cyrenaica* Maire & Weiller |  | Asteraceae | 1 | 0.68 | 0.6 | 0.2 | 0.4 | 0.15 | 2.03 | VPR | l | 2 | 1 | HP | no | P | 200 | 4.84 | no | KRI |
| *Cynoglossum sphacioticum* Boiss. & Heldr. |  | Boraginaceae | 0 | 0.8 | 0.4 | 0.2 | 0.4 | 0.3 | 2.10 | B | s | 2 | 4 | HP | no | H | 1700 | 13.97 | yes | KRI |
| *Dactylorhiza incarnata* (L.) Soó |  | Orchidaceae | 0 | 1.35 | 0.4 | 0.6 | 0.4 | 0.3 | 3.05 | VPR | m | 1 | 2 | G | yes | A | 740 | 341.93 | no | GRC |
| *Dactylorhiza kalopissii* E. Nelson subsp. *kalopissii* | *Dactylorhiza kalopissii* E. Nelson | Orchidaceae | 1 | 1.35 | 0.6 | 0.6 | 0.4 | 0.3 | 3.25 | VPR | m | 2 | 3 | G | yes | A | 1100 | 119.38 | yes | GRC |
| *Dactylorhiza kalopissii* subsp. *macedonica* (J. Hölz. & Künkele) Kreutz | *Dactylorhiza macedonica*J. Hölzinger & Künkele | Orchidaceae | 0 | 1.35 | 0.4 | 0.6 | 0.4 | 0.3 | 3.05 | VPR | m | 2 | 3 | G | yes | A | 600 | 231.01 | yes | GRC |
| *Dactylorhiza kalopissii* subsp. *pythagorae* (Gölz & H.R. Reinhard) Kreutz | *Dactylorhiza pythagorae* Gölz & H. R. Reinhard | Orchidaceae | 1 | 1.35 | 0.6 | 0.6 | 0.4 | 0.3 | 3.25 | VPR | l | 2 | 3 | G | yes | A | 800 | 0 | yes | EAI |
| *Damasonium bourgaei* Coss. | *Damasonium alisma* Miller | Alismataceae | 0 | 0.3 | 0.2 | 0.2 | 0.3 | 0.15 | 1.15 | W | s | 2 | 4 | HP | no | A | 0 | 170.53 | no | GRC |
| *Dianthus androsaceus* (Boiss. & Heldr.) Hayek |  | Caryophyllaceae | 0 | 0.83 | 0.6 | 0.2 | 0.3 | 0.15 | 2.08 | VPR | m | 2 | 2 | WP | no | H | 1000 | 139.23 | yes | GRC |
| *Dianthus arpadianus* Ade & Bornm. |  | Caryophyllaceae | 1 | 0.83 | 0.6 | 0.2 | 0.3 | 0.15 | 2.08 | VPR | s | 2 | 2 | HP | no | P | 300 | 48.07 | yes | GRC |
| *Dianthus haematocalyx* subsp*. phitosianus* Constantin. |  | Caryophyllaceae | 0 | 0.83 | 0.6 | 0.2 | 0.3 | 0.15 | 2.08 | VPR | l | 1 | 2 | HP | no | G | 10 | 21.4 | yes | GRC |
| *Dianthus ingoldbyi* Turrill |  | Caryophyllaceae | 1 | 0.83 | 0.6 | 0.2 | 0.3 | 0.15 | 2.08 | W | s | 2 | 3 | HP | no | M | 2 | 0 | yes | GRC |
| *Dianthus juniperinus* subsp. *kavusicus* Turland |  | Caryophyllaceae | 1 | 0.83 | 0.6 | 0.2 | 0.3 | 0.3 | 2.23 | VPR | m | 2 | 5 | HP | no | C | 240 | 8.75 | yes | KRI |
| *Dianthus simulans* Stoj. & Stef*.* |  | Caryophyllaceae | 0 | 0.83 | 0.6 | 0.2 | 0.3 | 0.15 | 2.08 | VPR | m | 2 | 2 | HP | no | H | 2000 | 0 | yes | GRC |
| *Dianthus xylorrhizus* Boiss. & Heldr. |  | Caryophyllaceae | 0 | 0.83 | 0.6 | 0.2 | 0.3 | 0.15 | 2.08 | W | m | 1 | 3 | HP | no | C | 250 | 3.23 | yes | KRI |
| *Dichoropetalum achaicum* (Halácsy) Pimenov & Kljuykov | *Peucedanum achaicum* Halácsy | Apiaceae | 0 | 0.3 | 0.2 | 0.2 | 0.3 | 0.15 | 1.15 | W | l | 2 | 2 | HP | no | G | 550 | 0 | yes | GRC |
| *Draba lacónica* Stevanović & Kit Tan |  | Brassicaceae | 1 | 0.83 | 0.2 | 0.2 | 0.3 | 0.3 | 1.83 | Ye | m | 1 | 4 | HP | no | H | 1500 | 8.17 | yes | GRC |
| *Draba nuda* (Bél.) Al-Shebaz & M. Koch |  | Brassicaceae | 0 | 0.83 | 0.2 | 0.2 | 0.3 | 0.3 | 1.83 | Ye | s | 1 | 1 | T | no | H | 1800 | 12.28 | no | KRI |
| *Draba strasseri* Greuter |  | Brassicaceae | 0 | 0.83 | 0.2 | 0.2 | 0.3 | 0.3 | 1.83 | Ye | s | 1 | 2 | HP | no | G | 700 | 12.32 | yes | GRC |
| *Drosera rotundifolia* L. |  | Droseraceae | 1 | 0.3 | 0.2 | 0.2 | 0.3 | 0.15 | 1.15 | W | m | 2 | 2 | HP | yes | A | 1140 | 6.32 | no | GRC |
| *Dryas octopetala* L. |  | Rosaceae | 0 | 0.3 | 0.2 | 0.2 | 0.3 | 0.15 | 1.15 | W | l | 2 | 2 | WP | yes | H | 1900 | 160.14 | no | GRC |
| *Drymocallis halacsyana*(Degen) Kurtto & Strid |  | Rosaceae | 1 | 0.3 | 0.2 | 0.2 | 0.3 | 0.15 | 1.15 | W | m | 2 | 1 | HP | yes | C | 1400 | 0 | yes | GRC |
| *Epilobium vernonicum* Snogerup |  | Onagraceae | 0 | 0.3 | 0.2 | 0.2 | 0.3 | 0.15 | 1.15 | VPR | m | 2 | 3 | HP | yes | A | 1500 | 86.78 | yes | GRC |
| *Epipactis cretica* Kalop. & Robatsch |  | Orchidaceae | 1 | 1.35 | 0.2 | 0.6 | 0.4 | 0.3 | 2.85 | Gr | s | 2 | 2 | G | yes | W | 700 | 160.53 | yes | KRI |
| *Epipactis greuteri* H. Baumann & Künkele |  | Orchidaceae | 1 | 1.35 | 0.2 | 0.6 | 0.4 | 0.3 | 2.85 | Gr | m | 2 | 2 | G | yes | W | 1100 | 277.76 | no | GRC |
| *Epipactis leptochila* subsp. *naousaensis* (Robatsch) Kreutz | *Epipactis naousaensis* Robatsch | Orchidaceae | 1 | 1.35 | 0.2 | 0.6 | 0.4 | 0.3 | 2.85 | VPR | s | 2 | 2 | G | yes | W | 1100 | 0 | yes | GRC |
| *Epipactis pontica* Taubenheim |  | Orchidaceae | 0 | 1.35 | 0.2 | 0.6 | 0.4 | 0.3 | 2.85 | Gr | s | 2 | 1 | G | yes | W | 200 | 0 | no | GRC |
| *Epipactis subclausa* Robatsch |  | Orchidaceae | 0 | 1.35 | 0.2 | 0.6 | 0.4 | 0.3 | 2.85 | Gr | s | 2 | 2 | G | yes | W | 700 | 352.49 | yes | GRC |
| *Eryngium amorginum* Rech. f. |  | Apiaceae | 0 | 0.68 | 0.2 | 0.2 | 0.4 | 0.15 | 1.63 | W | l | 2 | 3 | HP | no | C | 150 | 200.18 | yes | GRC |
| *Eryngium ternatum* Poir. |  | Apiaceae | 0 | 0.68 | 0.2 | 0.2 | 0.4 | 0.15 | 1.63 | W | m | 2 | 3 | HP | no | C | 100 | 120.22 | yes | KRI |
| *Erysimum krendlii* Polatschek |  | Brassicaceae | 0 | 0.83 | 0.4 | 0.2 | 0.3 | 0.3 | 2.03 | Ye | m | 2 | 3 | HP | no | P | 250 | 0 | yes | GRC |
| *Erysimum naxense* Snogerup |  | Brassicaceae | 0 | 0.83 | 0.4 | 0.2 | 0.3 | 0.3 | 2.03 | Ye | l | 1 | 3 | HP | no | C | 500 | 9.27 | yes | GRC |
| *Erysimum senoneri* subsp. *amorginum* Snogerup |  | Brassicaceae | 0 | 0.83 | 0.4 | 0.2 | 0.3 | 0.3 | 2.03 | Ye | m | 1 | 3 | WP | no | P | 150 | 17.17 | yes | GRC |
| *Erysimum senoneri* subsp. *icaricum* Snogerup |  | Brassicaceae | 0 | 0.83 | 0.4 | 0.2 | 0.3 | 0.3 | 2.03 | Ye | m | 1 | 3 | WP | no | C | 250 | 0 | yes | EAI |
| *Erythronium dens-canis* L. |  | Liliaceae | 0 | 1,00 | 0.2 | 0.2 | 0.3 | 0.15 | 1.85 | VPR | l | 1 | 2 | G | yes | H | 1200 | 72.17 | no | GRC |
| *Euphorbia rechingeri* Greuter |  | Euphorbiaceae | 0 | 0.3 | 0.2 | 0.2 | 0.3 | 0.15 | 1.15 | VPR | s | 1 | 2 | HP | no | H | 1750 | 6.2 | yes | KRI |
| *Euphorbia sultan-hassei* Strid & al. |  | Euphorbiaceae | 0 | 0.3 | 0.2 | 0.2 | 0.3 | 0.15 | 1.15 | Ye | s | 1 | 1 | WP | no | C | 0 | 37.64 | yes | KRI |
| *Ferulago sartorii* Boiss. |  | Apiaceae | 0 | 0.3 | 0.2 | 0.2 | 0.3 | 0.15 | 1.15 | Ye | l | 2 | 3 | HP | no | P | 200 | 0 | yes | GRC |
| *Fritillaria carica* Rix subsp. *carica* | *Fritillaria pelinaea* Kamari | Liliaceae | 1 | 1,00 | 0.6 | 0.2 | 0.3 | 0.15 | 2.25 | Ye | l | 1 | 2 | G | yes | P | 500 | 0 | yes | EAI |
| *Fritillaria conica* Boiss. |  | Liliaceae | 1 | 1,00 | 0.2 | 0.2 | 0.3 | 0.15 | 1.85 | Ye | l | 1 | 2 | G | yes | P | 0 | 49.18 | yes | GRC |
| *Fritillaria elwesii* Boiss. |  | Liliaceae | 1 | 1,00 | 0.6 | 0.2 | 0.3 | 0.15 | 2.25 | VPR | l | 1 | 2 | G | yes | P | 5 | 0 | no | EAI |
| *Fritillaria epirotica* Rix |  | Liliaceae | 0 | 1,00 | 0.2 | 0.2 | 0.3 | 0.15 | 1.85 | VPR | l | 1 | 2 | G | yes | H | 1600 | 72.52 | yes | GRC |
| *Fritillaria euboeica* Rix |  | Liliaceae | 0 | 1,00 | 0.6 | 0.2 | 0.3 | 0.15 | 2.25 | Ye | l | 1 | 3 | G | yes | W | 100 | 70.77 | yes | GRC |
| *Fritillaria obliqua* Ker-Gawl. subsp. *obliqua* |  | Liliaceae | 0 | 1,00 | 0.2 | 0.2 | 0.3 | 0.15 | 1.85 | VPR | l | 1 | 3 | G | yes | P | 0 | 63.43 | yes | GRC |
| *Fritillaria obliqua* subsp*. tuntasia* (Halácsy) Kamari |  | Liliaceae | 0 | 1,00 | 0.2 | 0.2 | 0.3 | 0.15 | 1.85 | VPR | l | 1 | 3 | G | yes | P | 20 | 107.62 | yes | GRC |
| *Fritillaria rhodia* A. Hansen |  | Liliaceae | 0 | 1,00 | 0.6 | 0.2 | 0.3 | 0.15 | 2.25 | Ye | m | 1 | 2 | G | yes | W | 0 | 55.3 | yes | EAI |
| *Fritillaria rhodokanakis* Orph. ex Baker |  | Liliaceae | 0 | 1,00 | 0.2 | 0.2 | 0.3 | 0.15 | 1.85 | VPR | l | 1 | 3 | G | yes | P | 10 | 30.25 | yes | GRC |
| *Fritillaria thessala* subsp. *reiseri* Kamari |  | Liliaceae | 0 | 1,00 | 0.6 | 0.2 | 0.3 | 0.15 | 2.25 | VPR | l | 1 | 2 | G | yes | P | 0 | 80.6 | yes | GRC |
| *Galanthus ikariae* Baker |  | Amaryllidaceae | 0 | 1,00 | 0.6 | 0.2 | 0.3 | 0.15 | 2.25 | W | l | 1 | 2 | G | yes | W | 500 | 223.82 | yes | GRC |
| *Galanthus nivalis* L. |  | Amaryllidaceae | 0 | 1,00 | 0.6 | 0.2 | 0.3 | 0.15 | 2.25 | W | l | 1 | 3 | G | yes | W | 100 | 327.95 | no | GRC |
| *Galanthus reginae-olgae* Orph. |  | Amaryllidaceae | 0 | 1,00 | 0.6 | 0.2 | 0.3 | 0.15 | 2.25 | W | l | 3 | 6 | G | yes | W | 50 | 348.72 | no | GRC |
| *Geocaryum bornmuelleri* (H. Wolff) Engstrand |  | Apiaceae | 1 | 0.3 | 0.2 | 0.2 | 0.3 | 0.15 | 1.15 | W | l | 1 | 3 | G | yes | R | 220 | 0 | yes | GRC |
| *Geocaryum creticum* (Boiss. & Heldr.) Engstrand |  | Apiaceae | 0 | 0.3 | 0.2 | 0.2 | 0.3 | 0.15 | 1.15 | W | l | 2 | 3 | G | yes | H | 1300 | 83.85 | yes | KRI |
| *Geocaryum divaricatum* (Boiss. & Heldr.) Engstrand |  | Apiaceae | 1 | 0.3 | 0.2 | 0.2 | 0.3 | 0.15 | 1.15 | W | l | 1 | 2 | G | yes | W | 1000 | 0 | yes | GRC |
| *Geranium thessalum* Franzén |  | Geraniaceae | 0 | 0.3 | 0.2 | 0.2 | 0.3 | 0.15 | 1.15 | VPR | l | 2 | 2 | HP | no | H | 1670 | 357.94 | yes | GRC |
| *Globularia stygia* Orph. ex Boiss. |  | Plantaginaceae | 0 | 0.68 | 0.2 | 0.2 | 0.4 | 0.15 | 1.63 | B | m | 2 | 2 | HP | yes | C | 1300 | 94.55 | yes | GRC |
| *Gymnadenia frivaldii* Griseb. | *Pseudorchis frivaldii* (Hampe ex Griseb.) P. F. Hunt | Orchidaceae | 0 | 1.35 | 0.2 | 0.6 | 0.4 | 0.3 | 2.85 | VPR | s | 2 | 2 | G | yes | A | 1000 | 268.4 | no | GRC |
| *Gymnadenia rhellicani* (Teppner & E. Klein) Teppner & E. Klein |  | Orchidaceae | 0 | 1.35 | 0.2 | 0.6 | 0.4 | 0.3 | 2.85 | VPR | s | 2 | 2 | G | yes | H | 1600 | 196.81 | no | GRC |
| *Gymnospermium peloponnesiacum* (Phitos) Strid | *Gymnospermium altaicum* subsp. *peloponnesiacum* Phitos | Berberidaceae | 0 | 0.3 | 0.2 | 0.2 | 0.3 | 0.3 | 1.30 | Ye | m | 1 | 3 | G | yes | G | 1300 | 74.82 | yes | GRC |
| *Haberlea rhodopensis* Friv. |  | Gesneriaceae | 0 | 1.13 | 0.6 | 0.6 | 0.4 | 0.15 | 2.88 | VPR | l | 2 | 4 | HP | yes | C | 0 | 133.39 | yes | GRC |
| *Haplophyllum megalanthum* Bornm. |  | Rutaceae | 0 | 0.3 | 0.2 | 0.2 | 0.3 | 0.15 | 1.15 | W | l | 1 | 2 | HP | no | P | 500 | 0 | yes | EAI |
| *Hedysarum grandiflorum* Pall. |  | Fabaceae | 1 | 1.28 | 0.6 | 0.6 | 0.4 | 0.3 | 3.18 | Ye | l | 1 | 2 | HP | no | G | 350 | 107.48 | no | GRC |
| *Helichrysum amorginum* Boiss. & Orph. |  | Asteraceae | 0 | 0.68 | 0.4 | 0.2 | 0.4 | 0.15 | 1.83 | W | m | 1 | 2 | HP | no | C | 83 | 44.57 | yes | GRC |
| *Helichrysum doerfleri* Rech. f. |  | Asteraceae | 1 | 0.68 | 0.4 | 0.2 | 0.4 | 0.15 | 1.83 | Ye | m | 1 | 2 | WP | no | P | 800 | 9.29 | yes | KRI |
| *Helichrysum heldreichii* Boiss. |  | Asteraceae | 0 | 0.68 | 0.4 | 0.2 | 0.4 | 0.15 | 1.83 | Ye | s | 1 | 2 | WP | no | C | 0 | 15.3 | yes | KRI |
| *Helichrysum sibthorpii* Rouy |  | Asteraceae | 1 | 0.68 | 0.4 | 0.2 | 0.4 | 0.15 | 1.83 | Ye | m | 2 | 4 | HP | no | C | 1840 | 0 | yes | GRC |
| *Helichrysum taenari* Rothm. |  | Asteraceae | 0 | 0.68 | 0.2 | 0.2 | 0.4 | 0.15 | 1.63 | Ye | s | 1 | 2 | HP | no | C | 200 | 62.24 | yes | GRC |
| *Himantoglossum comperianum* (Steven) P. Delforge | *Comperia comperiana* (Steven) Asch. & Graebn. | Orchidaceae | 0 | 1.35 | 0.2 | 0.6 | 0.4 | 0.3 | 2.85 | VPR | l | 2 | 3 | G | yes | H | 500 | 336.86 | no | EAI |
| *Horstrissea dolinicola* Greuter, P. Gerstberger & B. Egli |  | Apiaceae | 1 | 0.3 | 0.2 | 0.2 | 0.3 | 0.15 | 1.15 | VPR | m | 2 | 2 | G | yes | H | 1500 | 0 | yes | KRI |
| *Hyacinthella leucophaea* subsp. *atchleyi* (A.K. Jacks. & Turrill) K. M. Perss. & Jim. Perss. |  | Asparagaceae | 0 | 1,00 | 0.4 | 0.2 | 0.4 | 0.3 | 2.30 | B | s | 1 | 2 | G | yes | P | 100 | 355.34 | no | GRC |
| *Hypericum aciferum* (Greuter) N. Robson |  | Hypericaceae | 0 | 0.8 | 0.4 | 0.2 | 0.3 | 0.15 | 1.85 | Ye | s | 2 | 5 | WP | no | C | 4 | 0 | yes | KRI |
| *Hypericum fragile* Heldr. & Sartori ex Boiss. |  | Hypericaceae | 0 | 0.3 | 0.2 | 0.2 | 0.3 | 0.15 | 1.15 | Ye | l | 2 | 2 | HP | no | C | 5 | 59.88 | yes | GRC |
| *Hypericum jovis* Greuter |  | Hypericaceae | 0 | 0.3 | 0.2 | 0.2 | 0.3 | 0.15 | 1.15 | Ye | m | 1 | 2 | WP | no | C | 550 | 44.66 | yes | KRI |
| *Hypericum kelleri* Bald. |  | Hypericaceae | 0 | 0.3 | 0.2 | 0.2 | 0.3 | 0.15 | 1.15 | Ye | m | 2 | 4 | HP | yes | P | 1050 | 41.55 | yes | KRI |
| *Hypericum taygeteum* Quézel & Contandr. |  | Hypericaceae | 0 | 0.3 | 0.2 | 0.2 | 0.3 | 0.15 | 1.15 | Ye | m | 1 | 2 | HP | no | C | 400 | 55.97 | yes | GRC |
| *Iberis runemarkii* Greuter & Burdet |  | Brassicaceae | 0 | 0.68 | 0.4 | 0.2 | 0.3 | 0.15 | 1.73 | W | l | 1 | 2 | HP | no | C | 600 | 0 | yes | EAI |
| *Inula subfloccosa* Rech. f. |  | Asteraceae | 0 | 0.68 | 0.6 | 0.2 | 0.4 | 0.15 | 2.03 | Ye | l | 2 | 3 | HP | no | C | 250 | 6.58 | yes | GRC |
| *Isatis tinctoria* subsp. *athoa* (Boiss.) Papan. |  | Brassicaceae | 0 | 0.3 | 0.2 | 0.2 | 0.3 | 0.3 | 1.30 | Ye | s | 1 | 4 | HP | no | G | 1500 | 0 | yes | GRC |
| *Jankaea heldreichii* (Boiss.) Boiss. |  | Gesneriaceae | 0 | 1,00 | 0.4 | 0.2 | 0.4 | 0.15 | 2.15 | VPR | m | 2 | 4 | HP | no | C | 700 | 0 | yes | GRC |
| *Jurinea taygetea* Halácsy |  | Asteraceae | 1 | 0.68 | 0.6 | 0.2 | 0.4 | 0.15 | 2.03 | VPR | l | 2 | 3 | HP | no | H | 2000 | 0 | yes | GRC |
| *Lathraea rhodopea* Dingler |  | Orobanchaceae | 0 | 1.13 | 0.4 | 0.6 | 0.4 | 0.3 | 2.83 | VPR | s | 1 | 2 | G | yes | W | 100 | 156.4 | yes | GRC |
| *Lathraea squamaria* L. |  | Orobanchaceae | 0 | 1.13 | 0.6 | 0.6 | 0.4 | 0.3 | 3.03 | VPR | m | 1 | 3 | G | yes | W | 750 | 324.24 | no | GRC |
| *Lathyrus neurolobus* Boiss. & Heldr. |  | Fabaceae | 0 | 1.28 | 0.4 | 0.6 | 0.3 | 0.15 | 2.73 | B | s | 2 | 3 | HP | no | A | 180 | 30.38 | yes | KRI |
| *Lavandula pedunculata* subsp. *cariensis* (Boiss.) Upson & S. Andrews | *Lavandula cariensis* Boiss. | Lamiaceae | 0 | 1.13 | 0.4 | 0.6 | 0.4 | 0.3 | 2.83 | VPR | s | 1 | 4 | WP | no | P | 50 | 62.38 | yes | EAI |
| *Leontice leontopetalum* L.subsp. *leontopetalum* |  | Berberidaceae | 0 | 0.3 | 0.2 | 0.2 | 0.3 | 0.3 | 1.30 | Ye | m | 1 | 3 | G | yes | R | 0 | 749.22 | no | GRC |
| *Leontodon hellenicus* Phitos |  | Asteraceae | 0 | 0.68 | 0.6 | 0.2 | 0.4 | 0.15 | 2.03 | Ye | m | 2 | 2 | HP | no | C | 1750 | 103.97 | yes | GRC |
| *Lesquereuxia syriaca* Boiss. & Reut. |  | Orobanchaceae | 0 | 1.13 | 0.6 | 0.6 | 0.4 | 0.3 | 3.03 | VPR | l | 2 | 2 | HP | no | W | 300 | 213.31 | no | GRC |
| *Leucojum aestivum* L. |  | Amaryllidaceae | 0 | 1,00 | 0.2 | 0.2 | 0.3 | 0.15 | 1.85 | W | m | 1 | 3 | G | yes | A | 0 | 450.58 | no | GRC |
| *Lilium candidum* L. |  | Liliaceae | 0 | 0.8 | 0.2 | 0.2 | 0.3 | 0.15 | 1.65 | W | l | 1 | 2 | G | yes | G | 10 | 830.25 | no | GRC |
| *Lilium rhodopaeum* Delip. |  | Liliaceae | 0 | 1,00 | 0.2 | 0.2 | 0.3 | 0.15 | 1.85 | Ye | l | 2 | 2 | G | yes | H | 1200 | 13.11 | yes | GRC |
| *Limonium aphroditae* R. Artelari & Georgiou |  | Plumbaginaceae | 1 | 0.83 | 0.4 | 0.2 | 0.4 | 0.3 | 2.13 | VPR | s | 2 | 4 | HP | no | M | 0 | 0 | yes | GRC |
| *Limonium calliopsium* Alf. Mayer |  | Plumbaginaceae | 1 | 0.83 | 0.4 | 0.2 | 0.4 | 0.3 | 2.13 | B | s | 2 | 5 | HP | no | M | 0 | 0 | yes | KRI |
| *Limonium corinthiacum* (Boiss. & Heldr.) Kuntze |  | Plumbaginaceae | 1 | 0.83 | 0.2 | 0.2 | 0.4 | 0.3 | 1.93 | VPR | s | 2 | 4 | HP | no | M | 0 | 0 | yes | GRC |
| *Limonium cornarianum* Kypriot. & R. Artelari |  | Plumbaginaceae | 0 | 0.83 | 0.2 | 0.2 | 0.4 | 0.3 | 1.93 | VPR | s | 2 | 4 | HP | no | C | 0 | 0 | yes | KRI |
| *Limonium creticum* R. Artelari |  | Plumbaginaceae | 0 | 0.83 | 0.4 | 0.2 | 0.4 | 0.3 | 2.13 | VPR | s | 2 | 5 | HP | no | M | 0 | 0 | yes | KRI |
| *Limonium cythereum* R. Artelari & Georgiou |  | Plumbaginaceae | 0 | 0.83 | 0.4 | 0.2 | 0.4 | 0.3 | 2.13 | VPR | s | 2 | 4 | HP | no | M | 0 | 10.36 | yes | GRC |
| *Limonium damboldtianum* Phitos & R. Artelari |  | Plumbaginaceae | 0 | 0.83 | 0.4 | 0.2 | 0.4 | 0.3 | 2.13 | VPR | s | 2 | 4 | HP | no | M | 3 | 0 | yes | GRC |
| *Limonium elaphonisicum* Alf. Mayer |  | Plumbaginaceae | 0 | 0.83 | 0.4 | 0.2 | 0.4 | 0.3 | 2.13 | B | s | 2 | 5 | HP | no | M | 0 | 0 | yes | KRI |
| *Limonium glomeratum* (Tausch) Degen | *Limonium densiflorum* (Guss.) Kuntze | Plumbaginaceae | 0 | 0.83 | 0.4 | 0.2 | 0.4 | 0.3 | 2.13 | VPR | s | 2 | 5 | HP | no | M | 0 | 0 | no | GRC |
| *Limonium ithacense* R. Artelari |  | Plumbaginaceae | 0 | 0.83 | 0.4 | 0.2 | 0.4 | 0.3 | 2.13 | VPR | s | 2 | 3 | HP | no | M | 0 | 0 | yes | GRC |
| *Limonium kardamylii* R. Artelari & Kamari |  | Plumbaginaceae | 0 | 0.83 | 0.4 | 0.2 | 0.4 | 0.3 | 2.13 | VPR | s | 2 | 4 | HP | no | M | 0 | 0 | yes | GRC |
| *Limonium messeniacum* R. Artelari & Kamari |  | Plumbaginaceae | 1 | 0.83 | 0.4 | 0.2 | 0.4 | 0.3 | 2.13 | VPR | s | 2 | 4 | HP | no | M | 0 | 0 | yes | GRC |
| *Limonium phitosianum* R. Artelari |  | Plumbaginaceae | 0 | 0.83 | 0.4 | 0.2 | 0.4 | 0.3 | 2.13 | VPR | s | 2 | 3 | HP | no | M | 0 | 31.84 | yes | GRC |
| *Limonium zacynthium* R. Artelari |  | Plumbaginaceae | 0 | 0.83 | 0.4 | 0.2 | 0.4 | 0.3 | 2.13 | VPR | s | 2 | 3 | HP | no | M | 0 | 25.89 | yes | GRC |
| *Linaria tenuis* (Viv.) Spreng. |  | Plantaginaceae | 1 | 1.13 | 0.4 | 0.6 | 0.4 | 0.3 | 2.83 | Ye | m | 1 | 2 | T | no | R | 0 | 9.37 | no | GRC |
| *Linum hellenicum* Iatroú |  | Linaceae | 0 | 0.3 | 0.2 | 0.2 | 0.3 | 0.15 | 1.15 | VPR | l | 1 | 2 | HP | no | P | 400 | 27.76 | yes | GRC |
| *Linum phitosianum* Christod. & Iatroú |  | Linaceae | 1 | 0.8 | 0.4 | 0.2 | 0.3 | 0.15 | 1.85 | W | m | 1 | 2 | HP | no | P | 100 | 0 | yes | GRC |
| *Lithodora zahnii* (Halácsy) I.M. Johnst. |  | Boraginaceae | 0 | 0.8 | 0.4 | 0.2 | 0.4 | 0.15 | 1.95 | B | m | 1 | 4 | WP | no | C | 50 | 11.86 | yes | GRC |
| *Lomelosia minoana* subsp. *asterusica* (Greuter) Greuter & Burdet |  | Caprifoliaceae | 0 | 0.68 | 0.2 | 0.2 | 0.4 | 0.15 | 1.63 | VPR | l | 2 | 4 | WP | no | C | 1150 | 0 | yes | KRI |
| *Macrotomia densiflora* (Ledeb.) McBride | *Arnebia densiflora* (Ledeb.) Ledeb. | Boraginaceae | 0 | 0.8 | 0.6 | 0.2 | 0.4 | 0.3 | 2.30 | Ye | l | 2 | 2 | HP | no | C | 1200 | 64.01 | no | GRC |
| *Malus florentina* (Zucc.) C.K. Schneid. | X *Malosorbus florentina* (Zuccagni) Browicz | Rosaceae | 0 | 0.3 | 0.2 | 0.2 | 0.3 | 0.15 | 1.15 | W | m | 1 | 2 | WP | no | W | 900 | 443.21 | no | GRC |
| *Malus trilobata* (Poir.) C. K. Schneid. | *Eriolobus trilobatus* (Poiret) M. Roemer | Rosaceae | 0 | 0.3 | 0.2 | 0.2 | 0.3 | 0.15 | 1.15 | W | l | 1 | 2 | WP | no | W | 350 | 56.96 | no | GRC |
| *Medicago arborea* subsp. *strasseri* (Greuter, Matthäs & Risse) Sobr.-Vest. & Ceresuela | *Medicago strasseri* Greuter & al. | Fabaceae | 0 | 1.28 | 0.6 | 0.6 | 0.3 | 0.3 | 3.08 | Ye | s | 1 | 3 | WP | no | C | 0 | 123.36 | yes | KRI |
| *Medicago carica* (Hub.-Mor.) E. Small |  | Fabaceae | 0 | 1.28 | 0.4 | 0.6 | 0.3 | 0.15 | 2.73 | Ye | s | 1 | 2 | T | no | P | 10 | 6.22 | yes | EAI |
| *Medicago carstiensis* Wulfen |  | Fabaceae | 0 | 1.28 | 0.4 | 0.6 | 0.3 | 0.3 | 2.88 | Ye | s | 2 | 1 | HP | yes | W | 1050 | 0 | no | GRC |
| *Medicago heyniana* Greuter |  | Fabaceae | 0 | 1.28 | 0.4 | 0.6 | 0.3 | 0.15 | 2.73 | Ye | s | 1 | 1 | T | no | P | 500 | 221.24 | yes | EAI |
| *Medicago hypogaea* E. Small |  | Fabaceae | 0 | 1.28 | 0.2 | 0.6 | 0.3 | 0.15 | 2.53 | Ye | s | 1 | 1 | T | no | P | 100 | 8.98 | no | EAI |
| *Medicago muricoleptis* Tineo |  | Fabaceae | 0 | 1.28 | 0.4 | 0.6 | 0.3 | 0.15 | 2.73 | Ye | s | 1 | 2 | T | no | R | 0 | 242.81 | no | GRC |
| *Micromeria acropolitana* Halácsy | *Satureja acropolitana* (Halácsy) Greuter & Burdet | Lamiaceae | 1 | 1.13 | 0.2 | 0.6 | 0.4 | 0.3 | 2.63 | VPR | s | 1 | 2 | HP | no | C | 156 | 0 | yes | GRC |
| *Minuartia dirphya* Trigas & latroú |  | Caryophyllaceae | 1 | 1.05 | 0.2 | 0.2 | 0.3 | 0.15 | 1.90 | W | s | 2 | 5 | HP | no | P | 900 | 0 | yes | GRC |
| *Minuartia greuteriana* Kamari |  | Caryophyllaceae | 0 | 0.3 | 0.2 | 0.2 | 0.3 | 0.15 | 1.15 | W | s | 1 | 2 | HP | no | G | 100 | 10.33 | yes | GRC |
| *Minuartia parnonia* (Kamari) latroú, Trigas & Kit Tan |  | Caryophyllaceae | 0 | 1.05 | 0.2 | 0.2 | 0.3 | 0.15 | 1.90 | W | m | 2 | 5 | HP | no | G | 700 | 18.26 | yes | GRC |
| *Minuartia pichleri* (Boiss.) Maire & Petitm. |  | Caryophyllaceae | 0 | 0.8 | 0.2 | 0.2 | 0.3 | 0.15 | 1.65 | W | m | 1 | 3 | HP | no | C | 400 | 110.71 | yes | GRC |
| *Minuartia saxifraga* (Friv.) Graebn. subsp. *saxifraga* |  | Caryophyllaceae | 0 | 0.3 | 0.2 | 0.2 | 0.3 | 0.3 | 1.30 | W | m | 2 | 2 | HP | no | H | 900 | 155.7 | no | GRC |
| *Minuartia wettsteinii* Mattf. |  | Caryophyllaceae | 0 | 1.05 | 0.2 | 0.2 | 0.3 | 0.15 | 1.90 | W | s | 2 | 5 | HP | no | P | 1100 | 0 | yes | KRI |
| *Moluccella spinosa* L. |  | Lamiaceae | 0 | 1.13 | 0.6 | 0.6 | 0.4 | 0.3 | 3.03 | W | l | 1 | 4 | T | no | R | 40 | 497.87 | no | GRC |
| *Muscari kerkis* Karlén |  | Asparagaceae | 0 | 0.98 | 0.4 | 0.2 | 0.5 | 0.3 | 2.38 | VPR | s | 1 | 3 | G | yes | W | 600 | 0 | yes | EAI |
| *Myosotis solange* Greuter & Zaffran |  | Boraginaceae | 0 | 0.83 | 0.2 | 0.2 | 0.4 | 0.3 | 1.93 | B | s | 2 | 2 | HP | no | H | 2040 | 0 | yes | KRI |
| *Myosurus heldreichii* Heldr. ex H. Lév. |  | Ranunculaceae | 0 | 0.3 | 0.2 | 0.2 | 0.3 | 0.15 | 1.15 | Ye | s | 1 | 2 | T | no | A | 0 | 519.26 | no | EAI |
| *Neottia cordata* (L.) Rich. |  | Orchidaceae | 0 | 1.35 | 0.2 | 0.6 | 0.4 | 0.3 | 2.85 | Gr | s | 2 | 3 | G | yes | W | 1200 | 22.3 | no | GRC |
| *Nepeta sphaciotica* P.H. Davis |  | Lamiaceae | 1 | 1.13 | 0.4 | 0.6 | 0.4 | 0.3 | 2.83 | W | m | 2 | 1 | HP | no | H | 2200 | 0 | yes | KRI |
| *Noccaea cretica* (Degen & Jáv.) F. K. Mey. |  | Brassicaceae | 0 | 0.83 | 0.2 | 0.2 | 0.3 | 0.15 | 1.68 | W | s | 1 | 3 | HP | yes | H | 1450 | 151.41 | yes | KRI |
| *Noccaea zaffranii* (Greuter & Burdet) F.K. Mey. |  | Brassicaceae | 0 | 0.83 | 0.2 | 0.2 | 0.3 | 0.15 | 1.68 | W | s | 1 | 2 | HP | no | H | 1500 | 23.97 | yes | KRI |
| *Omphalodes runemarkii* Strid & Kit Tan | *Omphalodes verna* subsp*. graeca* Greuter | Boraginaceae | 0 | 0.83 | 0.2 | 0.2 | 0.4 | 0.15 | 1.78 | B | m | 1 | 1 | HP | yes | C | 700 | 14.98 | yes | GRC |
| *Onobrychis peloponnesiaca* (latroú & Kit Tan) latroú & Kit Tan |  | Fabaceae | 1 | 1.28 | 0.6 | 0.6 | 0.3 | 0.3 | 3.08 | Ye | m | 1 | 2 | HP | no | P | 100 | 0 | yes | GRC |
| *Onobrychis sphaciotica* Greuter |  | Fabaceae | 0 | 1.28 | 0.6 | 0.6 | 0.3 | 0.3 | 3.08 | VPR | m | 2 | 2 | HP | no | C | 1300 | 4.53 | yes | KRI |
| *Onosma elegantissima* Rech. f. & Goulimy |  | Boraginaceae | 0 | 0.98 | 0.6 | 0.2 | 0.5 | 0.3 | 2.58 | Ye | l | 2 | 3 | HP | no | G | 800 | 0 | yes | GRC |
| *Onosma sangiasensis* Teppner & Iatroú |  | Boraginaceae | 0 | 0.98 | 0.6 | 0.2 | 0.5 | 0.3 | 2.58 | Ye | m | 1 | 3 | HP | no | P | 350 | 28.49 | yes | GRC |
| *Onosma stridii* Teppner |  | Boraginaceae | 1 | 0.98 | 0.6 | 0.2 | 0.5 | 0.3 | 2.58 | W | l | 1 | 1 | HP | no | G | 300 | 30.74 | yes | GRC |
| *Ophrys argolica* H. Fleischm. |  | Orchidaceae | 0 | 1.35 | 0.2 | 0.6 | 0.4 | 0.3 | 2.85 | VPR | m | 1 | 2 | G | yes | W | 0 | 541.8 | no | GRC |
| *Ophrys helenae* Renz |  | Orchidaceae | 0 | 1.35 | 0.2 | 0.6 | 0.4 | 0.15 | 2.70 | VPR | m | 1 | 2 | G | yes | P | 0 | 351.12 | yes | GRC |
| *Ophrys scolopax* subsp. *rhodia* (H. Baumann & Künkele) H.A. Pedersen & Faurh. | *Ophrys umbilicata* subsp. *rhodia* H. Baumann & Künkele | Orchidaceae | 0 | 1.35 | 0.2 | 0.6 | 0.4 | 0.3 | 2.85 | VPR | s | 1 | 3 | G | yes | P | 0 | 240.57 | yes | EAI |
| *Orchis militaris* L. |  | Orchidaceae | 0 | 1.35 | 0.2 | 0.6 | 0.4 | 0.3 | 2.85 | VPR | m | 1 | 2 | G | yes | G | 1170 | 195.18 | no | GRC |
| *Orchis punctulata* Steven ex Lindl. |  | Orchidaceae | 1 | 1.35 | 0.2 | 0.6 | 0.4 | 0.3 | 2.85 | Ye | s | 1 | 2 | G | yes | P | 20 | 549.96 | no | GRC |
| *Orchis spitzelii* subsp. *nitidifolia* (W.P. Teschner) Soó | *Orchis prisca* Hautz. | Orchidaceae | 0 | 1.35 | 0.2 | 0.6 | 0.4 | 0.3 | 2.85 | VPR | m | 1 | 2 | G | yes | W | 700 | 191.87 | yes | KRI |
| *Origanum calcaratum* Juss. |  | Lamiaceae | 0 | 1.13 | 0.4 | 0.6 | 0.4 | 0.3 | 2.83 | VPR | m | 2 | 2 | HP | no | C | 0 | 202.44 | yes | GRC |
| *Origanum dictamnus* L. |  | Lamiaceae | 0 | 1.13 | 0.4 | 0.6 | 0.4 | 0.3 | 2.83 | VPR | s | 2 | 5 | HP | no | C | 0 | 211.69 | yes | KRI |
| *Origanum sipyleum* L. |  | Lamiaceae | 0 | 1.13 | 0.4 | 0.6 | 0.4 | 0.3 | 2.83 | VPR | m | 2 | 3 | HP | no | P | 470 | 112.52 | no | EAI |
| *Origanum symes* Carlström |  | Lamiaceae | 0 | 1.13 | 0.6 | 0.6 | 0.4 | 0.3 | 3.03 | VPR | m | 2 | 1 | HP | no | C | 0 | 0 | yes | EAI |
| *Origanum vetteri* Briq. & Barbey |  | Lamiaceae | 0 | 1.13 | 0.4 | 0.6 | 0.4 | 0.3 | 2.83 | VPR | s | 2 | 2 | HP | yes | C | 1100 | 3.78 | yes | KRI |
| *Origanum lirium* Heldr. ex Halácsy |  | Lamiaceae | 0 | 1.13 | 0.4 | 0.6 | 0.4 | 0.3 | 2.83 | VPR | m | 2 | 3 | G | yes | G | 1000 | 213.33 | yes | GRC |
| *Paeonia clusii* subsp. *rhodia* (Stearn) Tzanoud. |  | Paeoniaceae | 0 | 0.3 | 0.2 | 0.2 | 0.3 | 0.15 | 1.15 | W | l | 1 | 2 | G | yes | W | 300 | 33.82 | yes | EAI |
| *Paeonia corsica* Tausch | *Paeonia mascula* subsp. *russoi* (Biv.) Cullen & Heywood | Paeoniaceae | 0 | 0.3 | 0.2 | 0.2 | 0.3 | 0.15 | 1.15 | VPR | l | 1 | 2 | G | yes | W | 0 | 122.38 | no | GRC |
| *Paeonia parnassica* Tzanoud. |  | Paeoniaceae | 0 | 0.3 | 0.2 | 0.2 | 0.3 | 0.15 | 1.15 | VPR | l | 1 | 2 | G | yes | W | 800 | 47.64 | yes | GRC |
| *Paracaryum lithospermifolium* subsp. *cariense* (Boiss.) R.R. Mill |  | Boraginaceae | 0 | 0.8 | 0.2 | 0.2 | 0.4 | 0.3 | 1.90 | VPR | s | 2 | 3 | HP | no | H | 950 | 369.52 | no | KRI |
| *Paronychia bornmuelleri* Chaudhri |  | Caryophyllaceae | 0 | 0.3 | 0.2 | 0.2 | 0.3 | 0.3 | 1.30 | W | s | 1 | 2 | HP | no | P | 240 | 0 | yes | GRC |
| *Periploca angustifolia* Labill. |  | Apocynaceae | 0 | 0.3 | 0.2 | 0.2 | 0.3 | 0.3 | 1.30 | VPR | s | 1 | 2 | WP | no | P | 0 | 143.99 | no | KRI |
| *Petrorhagia grandiflora* Iatroú |  | Caryophyllaceae | 0 | 0.8 | 0.6 | 0.2 | 0.3 | 0.15 | 2.05 | W | l | 1 | 2 | HP | no | C | 50 | 20.65 | yes | GRC |
| *Petteria ramentacea* (Sieber) C. Presl |  | Fabaceae | 0 | 1.28 | 0.6 | 0.6 | 0.3 | 0.3 | 3.08 | Ye | l | 1 | 1 | WP | no | W | 115 | 0 | no | GRC |
| *Phelipanche schultzioides* M.J.Y. Foley | *Orobanche schultzioides* (M.J.Y.Foley) Domina | Orobanchaceae | 1 | 1.13 | 0.6 | 0.6 | 0.4 | 0.3 | 3.03 | B | m | 2 | 1 | T | no | R | 1000 | 59.03 | yes | GRC |
| *Phitosia crocifolia* (Boiss. & Heldr.) Kamari & Greuter |  | Asteraceae | 1 | 0.68 | 0.4 | 0.2 | 0.4 | 0.15 | 1.83 | Ye | m | 2 | 4 | HP | no | H | 1350 | 21.21 | yes | GRC |
| *Phoenix theophrasti* Greuter |  | Arecaceae | 0 | 0.3 | 0.2 | 0.6 | 0.3 | 0.3 | 1.70 | Ye | s | 1 | 3 | WP | no | A | 0 | 246.24 | no | KRI |
| *Pimpinella pretenderis* (Heldr.) Halácsy |  | Apiaceae | 0 | 0.3 | 0.2 | 0.2 | 0.3 | 0.15 | 1.15 | W | l | 1 | 2 | HP | no | C | 20 | 238.63 | yes | GRC |
| *Polygala helenae* Greuter |  | Polygalaceae | 1 | 1.28 | 0.4 | 0.6 | 0.4 | 0.3 | 2.98 | B | s | 1 | 2 | HP | no | P | 100 | 0 | yes | GRC |
| *Polygala subuniflora* Boiss. & Heldr. |  | Polygalaceae | 1 | 1.28 | 0.2 | 0.6 | 0.4 | 0.15 | 2.63 | B | s | 2 | 2 | HP | no | H | 2000 | 0 | yes | GRC |
| *Polygonum idaeum* Hayek |  | Polygonaceae | 0 | 0.3 | 0.2 | 0.2 | 0.4 | 0.15 | 1.25 | W | s | 2 | 4 | WP | yes | H | 1400 | 62.38 | yes | KRI |
| *Polygonum papillosum* Hartvig |  | Polygonaceae | 0 | 0.3 | 0.2 | 0.2 | 0.4 | 0.3 | 1.40 | W | s | 2 | 2 | HP | no | G | 600 | 61.48 | yes | GRC |
| *Polygonum praelongum* Coode & Cullen |  | Polygonaceae | 1 | 0.3 | 0.2 | 0.2 | 0.4 | 0.3 | 1.40 | VPR | s | 2 | 1 | HP | no | M | 15 | 0 | yes | EAI |
| *Potentilla arcadiensis* Iatroú |  | Rosaceae | 0 | 0.3 | 0.2 | 0.2 | 0.3 | 0.15 | 1.15 | W | m | 2 | 2 | HP | no | C | 450 | 32.9 | yes | GRC |
| *Potentilla kionaea* Halácsy |  | Rosaceae | 0 | 0.3 | 0.2 | 0.2 | 0.3 | 0.15 | 1.15 | VPR | s | 2 | 2 | HP | no | C | 2300 | 0 | yes | GRC |
| *Prometheum tymphaeum* (Quézel & Contandr.) 't Hart |  | Crassulaceae | 0 | 0.3 | 0.2 | 0.2 | 0.4 | 0.15 | 1.25 | W | m | 2 | 1 | HP | yes | H | 1700 | 63.15 | yes | GRC |
| *Pterocephalus brevis* Coult. |  | Caprifoliaceae | 0 | 0.68 | 0.4 | 0.2 | 0.4 | 0.15 | 1.83 | VPR | l | 1 | 2 | T | no | R | 0 | 6.57 | no | KRI |
| *Pulmonaria cesatiana* (Fenzl & Friedr.) Selvi & al. | *Nonea cesatiana* (Fenzl & Friedr.) Greuter & Burdet | Boraginaceae | 0 | 0.8 | 0.6 | 0.2 | 0.4 | 0.3 | 2.30 | VPR | m | 1 | 3 | HP | no | W | 1000 | 99.72 | yes | GRC |
| *Ramonda serbica* Pančić |  | Gesneriaceae | 0 | 0.3 | 0.2 | 0.2 | 0.4 | 0.15 | 1.25 | VPR | l | 1 | 3 | HP | yes | C | 400 | 124.05 | yes | GRC |
| *Ranunculus cacuminis* Strid & Papan. |  | Ranunculaceae | 0 | 0.3 | 0.2 | 0.2 | 0.3 | 0.15 | 1.15 | W | l | 2 | 3 | G | yes | H | 1900 | 0 | yes | GRC |
| *Ranunculus radinotrichus* Greuter & Strid |  | Ranunculaceae | 0 | 0.3 | 0.2 | 0.2 | 0.3 | 0.15 | 1.15 | Ye | m | 1 | 4 | G | yes | H | 1850 | 0 | yes | KRI |
| *Ranunculus veronicae* N. Böhling |  | Ranunculaceae | 1 | 0.3 | 0.2 | 0.2 | 0.3 | 0.15 | 1.15 | Ye | m | 1 | 3 | G | yes | C | 370 | 0 | yes | KRI |
| *Reseda odorata* L. |  | Resedaceae | 0 | 1.05 | 0.2 | 0.2 | 0.3 | 0.3 | 2.05 | W | s | 1 | 4 | T | no | R | 0 | 226.87 | no | KRI |
| *Rhaponticoides amplifolia* (Boiss. & Heldr.) M.V. Agab. & Greuter | *Centaurea amplifolia* Boiss. & Heldr*.* | Asteraceae | 1 | 0.68 | 0.6 | 0.2 | 0.4 | 0.15 | 2.03 | VPR | l | 2 | 1 | HP | no | G | 1300 | 0 | yes | GRC |
| *Rhododendron luteum* Sweet |  | Ericaceae | 0 | 0.8 | 0.2 | 0.2 | 0.4 | 0.3 | 1.90 | Ye | l | 1 | 2 | WP | yes | W | 60 | 8.39 | no | EAI |
| *Ricotia isatoides* (Barbey) B.L. Burtt |  | Brassicaceae | 0 | 0.83 | 0.2 | 0.2 | 0.3 | 0.3 | 1.83 | VPR | s | 1 | 2 | HP | no | P | 450 | 0 | yes | KRI |
| *Roemeria hybrida* (L.) DC. subsp. *hybrida* |  | Papaveraceae | 0 | 0.3 | 0.2 | 0.2 | 0.3 | 0.15 | 1.15 | VPR | l | 1 | 2 | T | no | R | 0 | 734.37 | no | GRC |
| *Rorippa icarica* Rech. f. |  | Brassicaceae | 0 | 0.83 | 0.4 | 0.2 | 0.3 | 0.3 | 2.03 | Ye | s | 1 | 3 | HP | no | C | 300 | 24.78 | yes | EAI |
| *Sagittaria sagittifolia* L. |  | Alismataceae | 0 | 0.3 | 0.2 | 0.2 | 0.3 | 0.15 | 1.15 | W | l | 2 | 2 | HP | yes | A | 0 | 278.42 | no | GRC |
| *Salix xanthicola* K.I. Chr. |  | Salicaceae | 0 | 1.05 | 0.2 | 0.2 | 0.3 | 0.3 | 2.05 | VPR | s | 1 | 1 | WP | no | A | 30 | 137.21 | yes | GRC |
| *Saponaria aenesia* Heldr. |  | Caryophyllaceae | 1 | 0.83 | 0.4 | 0.2 | 0.3 | 0.3 | 2.03 | VPR | s | 1 | 2 | T | no | G | 400 | 19.8 | yes | GRC |
| *Saponaria jagelii* Phitos & Greuter |  | Caryophyllaceae | 1 | 0.83 | 0.4 | 0.2 | 0.3 | 0.3 | 2.03 | VPR | m | 1 | 3 | T | no | M | 0 | 0 | yes | GRC |
| *Scorzonera mollis* subsp*. idaea* (Gand.) Lack |  | Asteraceae | 0 | 0.68 | 0.6 | 0.2 | 0.4 | 0.15 | 2.03 | Ye | m | 2 | 3 | G | yes | H | 1400 | 80.89 | yes | KRI |
| *Scorzonera scyria* M.A. Gust. & Snogerup |  | Asteraceae | 0 | 0.68 | 0.6 | 0.2 | 0.4 | 0.15 | 2.03 | Ye | l | 1 | 2 | HP | no | C | 50 | 0 | yes | GRC |
| *Scrophularia spinulescens* Hausskn. & Degen |  | Scrophulariaceae | 0 | 1.13 | 0.4 | 0.6 | 0.4 | 0.15 | 2.68 | VPR | s | 2 | 2 | HP | no | G | 800 | 0 | yes | GRC |
| *Scutellaria rupestris* subsp. *cephalonica* (Rech. f.) Greuter & Burdet |  | Lamiaceae | 1 | 1.13 | 0.6 | 0.6 | 0.4 | 0.3 | 3.03 | VPR | m | 2 | 3 | HP | no | C | 800 | 0 | yes | GRC |
| *Scutellaria rupestris* subsp. *rechingeri* Bothmer |  | Lamiaceae | 0 | 1.13 | 0.6 | 0.6 | 0.4 | 0.3 | 3.03 | VPR | m | 2 | 3 | HP | no | G | 1000 | 0 | yes | GRC |
| *Scutellaria rupestris* Boiss. & Heldr. subsp. *rupestris* |  | Lamiaceae | 0 | 1.13 | 0.6 | 0.6 | 0.4 | 0.3 | 3.03 | VPR | m | 2 | 2 | HP | no | H | 1800 | 1.92 | yes | GRC |
| *Sedum stefco* Stef. |  | Crassulaceae | 0 | 0.3 | 0.2 | 0.2 | 0.3 | 0.3 | 1.30 | W | s | 2 | 2 | HP | yes | C | 1000 | 250.37 | yes | GRC |
| *Senecio eubaeus* Boiss. & Heldr. |  | Asteraceae | 0 | 0.68 | 0.4 | 0.2 | 0.4 | 0.15 | 1.83 | Ye | l | 2 | 3 | HP | no | G | 900 | 42.16 | yes | GRC |
| *Sibthorpia europaea* L. |  | Plantaginaceae | 0 | 0.3 | 0.2 | 0.2 | 0.4 | 0.15 | 1.25 | W | s | 1 | 4 | HP | yes | A | 180 | 482.32 | no | KRI |
| *Sideritis euboea* Heldr. |  | Lamiaceae | 1 | 1.13 | 0.4 | 0.6 | 0.4 | 0.3 | 2.83 | Ye | s | 2 | 4 | HP | no | H | 600 | 83.41 | yes | GRC |
| *Sideritis raeseri* subsp. *attica* (Heldr.) Papan. & Kokkini |  | Lamiaceae | 0 | 1.13 | 0.6 | 0.6 | 0.4 | 0.3 | 3.03 | Ye | m | 2 | 2 | HP | no | G | 900 | 55.11 | yes | GRC |
| *Sideritis sipylea* Boiss. |  | Lamiaceae | 1 | 1.13 | 0.4 | 0.6 | 0.4 | 0.3 | 2.83 | Ye | s | 2 | 2 | HP | no | P | 250 | 165.59 | no | EAI |
| *Silene ammophila* Boiss. & Heldr. subsp. *ammophila* |  | Caryophyllaceae | 1 | 0.83 | 0.4 | 0.2 | 0.3 | 0.15 | 1.88 | VPR | s | 1 | 3 | T | no | M | 0 | 67.24 | yes | KRI |
| *Silene ammophila* subsp. *carpathae* Chowdhuri |  | Caryophyllaceae | 0 | 0.83 | 0.6 | 0.2 | 0.3 | 0.15 | 2.08 | VPR | m | 1 | 2 | T | no | M | 0 | 51.62 | yes | KRI |
| *Silene cephallenia* Heldr. subsp. *cephallenia* |  | Caryophyllaceae | 1 | 0.83 | 0.6 | 0.2 | 0.3 | 0.3 | 2.23 | W | m | 2 | 2 | HP | no | C | 20 | 159.66 | yes | GRC |
| *Silene flavescens* (Rech. f.) Greuter subsp. *dictaea* |  | Caryophyllaceae | 0 | 0.83 | 0.4 | 0.2 | 0.3 | 0.15 | 1.88 | Ye | m | 2 | 5 | HP | no | C | 1400 | 12.42 | yes | KRI |
| *Silene holzmannii* Heldr. ex Boiss*.* |  | Caryophyllaceae | 1 | 0.83 | 0.6 | 0.2 | 0.3 | 0.3 | 2.23 | VPR | s | 1 | 2 | T | no | M | 0 | 439.33 | yes | GRC |
| *Silene integripetala* subsp. *greuteri* (Phitos) Akeroyd |  | Caryophyllaceae | 0 | 0.83 | 0.6 | 0.2 | 0.3 | 0.3 | 2.23 | VPR | m | 1 | 3 | T | no | C | 150 | 82.95 | yes | KRI |
| *Silene orphanidis* Boiss. |  | Caryophyllaceae | 1 | 0.83 | 0.4 | 0.2 | 0.3 | 0.15 | 1.88 | W | m | 2 | 3 | HP | no | H | 1830 | 0 | yes | GRC |
| *Silene succulenta* Forssk. subsp. *succulenta* |  | Caryophyllaceae | 0 | 0.83 | 0.6 | 0.2 | 0.3 | 0.15 | 2.08 | W | m | 1 | 3 | HP | no | M | 0 | 238.87 | no | KRI |
| *Soldanella chrysosticta* subsp. *pelia* (Raus) Raus | *Soldanella pelia* Raus | Primulaceae | 0 | 1,00 | 0.2 | 0.2 | 0.4 | 0.15 | 1.95 | VPR | s | 1 | 2 | HP | no | A | 1200 | 0 | yes | GRC |
| *Soldanella rhodopaea* F.K. Mey*.* |  | Primulaceae | 0 | 1,00 | 0.2 | 0.2 | 0.4 | 0.15 | 1.95 | VPR | s | 1 | 4 | HP | no | W | 1100 | 46.16 | yes | GRC |
| *Solenanthus stamineus* (Desf.) Wettst. | *Cynoglossum stamineum* Desf. | Boraginaceae | 0 | 0.98 | 0.4 | 0.2 | 0.4 | 0.3 | 2.28 | VPR | s | 2 | 3 | HP | no | H | 1650 | 71.35 | no | GRC |
| *Sorbus umbellata* subsp. *baldaccii* (C.K. Schneid.) K.I. Chr. | *Sorbus baldaccii* (C. K. Schneid.) Zinserl. | Rosaceae | 0 | 0.3 | 0.2 | 0.2 | 0.3 | 0.15 | 1.15 | W | m | 2 | 1 | WP | no | W | 1350 | 253.66 | no | GRC |
| *Spiraea chamaedryfolia* L. |  | Rosaceae | 0 | 1.05 | 0.2 | 0.2 | 0.3 | 0.15 | 1.90 | W | m | 1 | 2 | WP | no | W | 700 | 47.1 | no | GRC |
| *Stachys euboica* Rech. f. |  | Lamiaceae | 0 | 1.13 | 0.6 | 0.6 | 0.4 | 0.3 | 3.03 | W | m | 1 | 2 | HP | no | C | 200 | 0 | yes | GRC |
| *Stachys spreitzenhoferi* subsp. *virella* D. Perss. |  | Lamiaceae | 1 | 1.13 | 0.4 | 0.6 | 0.4 | 0.3 | 2.83 | W | m | 1 | 2 | HP | no | C | 50 | 0 | yes | GRC |
| *Stachys swainsonii* subsp. *melangavica* D. Perss. |  | Lamiaceae | 0 | 1.13 | 0.4 | 0.6 | 0.4 | 0.3 | 2.83 | VPR | m | 1 | 3 | HP | no | C | 0 | 119.46 | yes | GRC |
| *Stachys swainsonii* subsp. *scyronica* (Boiss.) Phitos & Damboldt |  | Lamiaceae | 0 | 1.13 | 0.4 | 0.6 | 0.4 | 0.3 | 2.83 | W | m | 1 | 3 | HP | no | C | 750 | 3.25 | yes | GRC |
| *Symphytum davisii* subsp. *cycladense* (Pawl.) Stearn |  | Boraginaceae | 0 | 0.98 | 0.6 | 0.2 | 0.5 | 0.3 | 2.58 | W | m | 1 | 2 | HP | no | C | 90 | 6.06 | yes | GRC |
| *Teucrium aroanium* Orph. ex Boiss. |  | Lamiaceae | 0 | 1.13 | 0.2 | 0.6 | 0.4 | 0.3 | 2.63 | B | l | 2 | 4 | WP | no | C | 420 | 145.64 | yes | GRC |
| *Teucrium cuneifolium* Sm. |  | Lamiaceae | 0 | 1.13 | 0.4 | 0.6 | 0.4 | 0.15 | 2.68 | Ye | s | 1 | 1 | WP | no | C | 100 | 47.91 | yes | KRI |
| *Teucrium francisci-werneri* Rech. f. |  | Lamiaceae | 0 | 1.13 | 0.4 | 0.6 | 0.4 | 0.3 | 2.83 | VPR | m | 2 | 3 | HP | no | C | 20 | 110 | yes | GRC |
| *Teucrium montbretii* subsp. *heliotropiifolium* (Barbey) P.H. Davis |  | Lamiaceae | 0 | 1.13 | 0.2 | 0.6 | 0.4 | 0.3 | 2.63 | W | s | 2 | 1 | HP | no | C | 0 | 130.26 | yes | KRI |
| *Thesium vlachorum* Aldén |  | Santalaceae | 1 | 0.8 | 0.2 | 0.2 | 0.4 | 0.3 | 1.90 | W | s | 2 | 1 | HP | yes | H | 1900 | 0 | yes | GRC |
| *Thymbra calostachya* (Rech. f.) Rech. f. |  | Lamiaceae | 0 | 1.13 | 0.4 | 0.6 | 0.4 | 0.3 | 2.83 | W | s | 1 | 2 | WP | no | C | 0 | 7.4 | yes | KRI |
| *Thymus hartvigii* R. Morales subsp*. hartvigii* |  | Lamiaceae | 0 | 1.13 | 0.6 | 0.6 | 0.4 | 0.3 | 3.03 | VPR | m | 2 | 3 | HP | yes | H | 1600 | 81.53 | yes | GRC |
| *Thymus laconicus* Jalas |  | Lamiaceae | 0 | 1.13 | 0.4 | 0.6 | 0.4 | 0.3 | 2.83 | VPR | m | 1 | 2 | WP | no | P | 50 | 75.09 | yes | GRC |
| *Thymus plasonii* Adamović |  | Lamiaceae | 1 | 1.13 | 0.4 | 0.6 | 0.4 | 0.3 | 2.83 | VPR | s | 1 | 2 | HP | no | G | 63 | 292.09 | yes | GRC |
| *Thymus sipyleus* Boiss. |  | Lamiaceae | 0 | 1.13 | 0.4 | 0.6 | 0.4 | 0.15 | 2.68 | W | s | 2 | 2 | WP | no | H | 1000 | 105.52 | no | EAI |
| *Tragopogon lassithicus* Rech. f. |  | Asteraceae | 0 | 0.68 | 0.4 | 0.2 | 0.4 | 0.15 | 1.83 | Ye | m | 1 | 1 | HP | yes | H | 1800 | 67.52 | yes | KRI |
| *Tripleurospermum conoclinium* (Boiss. & Balansa) Hayek |  | Asteraceae | 1 | 0.68 | 0.2 | 0.2 | 0.4 | 0.15 | 1.63 | W | l | 1 | 4 | HP | no | G | 0 | 0 | no | EAI |
| *Tulipa goulimyi*Sealy & Turrill |  | Liliaceae | 0 | 0.8 | 0.2 | 0.2 | 0.3 | 0.15 | 1.65 | VPR | l | 1 | 2 | G | yes | P | 0 | 165.41 | yes | GRC |
| *Tulipa undulatifolia* Boiss. |  | Liliaceae | 0 | 0.8 | 0.2 | 0.2 | 0.3 | 0.15 | 1.65 | VPR | l | 1 | 2 | G | yes | R | 0 | 428.82 | no | GRC |
| *Utricularia australis* R. Br. |  | Lentibulariaceae | 0 | 1.13 | 0.4 | 0.6 | 0.4 | 0.15 | 2.68 | Ye | l | 2 | 1 | HP | yes | A | 0 | 400.92 | no | GRC |
| *Utricularia gibba* L. |  | Lentibulariaceae | 1 | 1.13 | 0.4 | 0.6 | 0.4 | 0.15 | 2.68 | Ye | l | 1 | 12 | HP | yes | A | 90 | 0 | no | GRC |
| *Valeriana crinii* Orph. ex Boiss. |  | Caprifoliaceae | 0 | 0.8 | 0.4 | 0.2 | 0.4 | 0.15 | 1.95 | W | s | 2 | 2 | HP | yes | H | 1200 | 296.8 | yes | GRC |
| *Verbascum cylleneum* (Boiss. & Heldr.) Kuntze |  | Scrophulariaceae | 0 | 0.83 | 0.2 | 0.2 | 0.4 | 0.15 | 1.78 | Ye | m | 2 | 3 | HP | no | H | 1900 | 0 | yes | GRC |
| *Verbascum syriacum* Schrad. |  | Scrophulariaceae | 1 | 0.83 | 0.2 | 0.2 | 0.4 | 0.3 | 1.93 | Ye | m | 1 | 2 | HP | no | M | 0 | 28.49 | yes | EAI |
| *Veronica oetaea* Gustavsson |  | Plantaginaceae | 1 | 0.3 | 0.2 | 0.6 | 0.4 | 0.3 | 1.80 | W | s | 1 | 2 | T | no | A | 1850 | 0 | yes | GRC |
| *Veronica stamatiadae* M.A. Fisch. & Greuter |  | Plantaginaceae | 0 | 0.83 | 0.2 | 0.6 | 0.4 | 0.3 | 2.33 | W | m | 1 | 2 | T | no | P | 20 | 0 | yes | EAI |
| *Vincetoxicum creticum* Browicz |  | Apocynaceae | 0 | 0.3 | 0.2 | 0.2 | 0.4 | 0.3 | 1.40 | Ye | s | 2 | 2 | HP | no | H | 1150 | 146.51 | yes | KRI |
| *Viola athois* W. Becker |  | Violaceae | 0 | 1.13 | 0.6 | 0.6 | 0.3 | 0.15 | 2.78 | VPR | l | 1 | 4 | HP | no | H | 1500 | 0 | yes | GRC |
| *Viola cephalonica* Bornm. |  | Violaceae | 1 | 1.13 | 0.6 | 0.6 | 0.3 | 0.15 | 2.78 | VPR | l | 1 | 2 | HP | no | C | 1600 | 0 | yes | GRC |
| *Viola oligyrtia* Tiniakou |  | Violaceae | 0 | 1.13 | 0.4 | 0.6 | 0.3 | 0.15 | 2.58 | VPR | m | 1 | 2 | HP | yes | G | 1100 | 0 | yes | GRC |
| *Viola scorpiuroides* Coss. |  | Violaceae | 0 | 1.13 | 0.4 | 0.6 | 0.3 | 0.15 | 2.58 | Ye | m | 1 | 5 | HP | no | P | 0 | 320.16 | no | KRI |
| *Viola striis-notata* (J. Wagner) Merxm. & Lippert |  | Violaceae | 0 | 1.13 | 0.4 | 0.6 | 0.3 | 0.15 | 2.58 | VPR | l | 2 | 2 | HP | no | H | 2400 | 0 | yes | GRC |

See Methods for details on each variable.

^1^The original taxon name appearing in the Greek Red Data Book, when this is other than the one used in this study due to nomenclatural update (see Methods).

^2^0: “Less threatened” (Vulnerable, Near Threatened, Rare and Least Concerned taxa*sensu* IUCN) and 1: “More threatened” (Critically Endangered, Endangered and presumably Extinct taxa *sensu* IUCN)

^3^ Floral Complexity Index

^4^ Corolla segmentation

^5^ Functional reproductive unit

^6^B: blue; Gr: green; VPR: violet–pink–purple–red; W: white; Ye: yellow

^7^s: small, m: medium, l: large

^8^G: geophytes; HP: herbaceous perennials; T: therophytes; WP: woody perennials

^9^ Asexual reproduction

^10^A: aquatic/freshwater; C: cliffs; G: lowland to montane grasslands; H: high-mountain; M: coastal and marine; P: phrygana; R: agricultural and ruderal; W: woodlands and scrub

^11^ Phytogeographical region. KRI: Kriti–Karpathos group; EAI: East Aegean Islands; GRC: rest of Greece.
