# Supplementary material for: Lessons from Red Data Books: Plant Vulnerability Increases with Floral Complexity
Source: PLoS One. 2015 Sep 21;10(9):e0138414. doi: 10.1371/journal.pone.0138414 (PMC4577097; doi:10.1371/journal.pone.0138414)
Supplement: S1 Text — (DOCX) [file pone.0138414.s002.docx]

**S1 Text. Floral variable level values.**

The values (*V*) assigned to the levels of the five floral variables comprising the Floral Complexity Index were the means of scores provided independently by four pollination ecology experts, namely Theodora Petanidou, Thomas Tscheulin, Aphrodite Kantsa and Jelle Devalez, outlining their opinion on the contribution of each of the five variables’ levels to floral complexity (Tables S1.1 to S1.5).

Table S1.1.Values assigned to the levels of the floral variable “shape”. The value (*V*) of each variable’s level in the index is the mean of four assigned scores.

| **Value (*V*)** | **bell** | **brush** | **disk** | **tube** | **disk-tube** | **funnel** | **flag** | **gullet** | **head** | **lip** | **trap** |
| --- | --- | --- | --- | --- | --- | --- | --- | --- | --- | --- | --- |
| Score 1 | 4 | 2 | 1 | 3 | 3 | 3 | 5 | 4 | 2 | 5 | 5 |
| Score 2 | 3 | 2 | 1 | 3 | 3 | 3 | 5 | 4 | 2 | 5 | 5 |
| Score 3 | 3 | 5 | 1 | 3 | 2 | 2 | 4 | 4 | 3 | 4 | 2 |
| Score 4 | 3.5 | 5 | 1 | 4 | 3 | 2.5 | 3 | 3 | 2 | 4 | 5 |
| **Mean** | **3.33** | **3.5** | **1** | **3.25** | **2.75** | **2.67** | **4.25** | **3.75** | **2.25** | **4.5** | **4.25** |

Table S1.2. Values assigned to the levels of the floral variable “depth”. The value (*V*) of each variable’s level in the index is the mean of four assigned scores.

| **Value (*V*)** | **low-depth** | **medium-depth** | **high-depth** |
| --- | --- | --- | --- |
| Score 1 | 1 | 2 | 3 |
| Score 2 | 1 | 2 | 3 |
| Score 3 | 1 | 2 | 3 |
| Score 4 | 1 | 2 | 3 |
| **Mean** | **1** | **2** | **3** |

Table S1.3. Values assigned to the levels of the floral variable “symmetry”. The value (*V*) of each variable’s level in the index is the mean of four assigned scores.

| **Value (*V*)** | **bilateral** | **radial** |
| --- | --- | --- |
| Score 1 | 3 | 1 |
| Score 2 | 3 | 1 |
| Score 3 | 3 | 1 |
| Score 4 | 3 | 1.5 |
| **Mean** | **3** | **1.13** |

Table S1.4. Values assigned to the levels of the floral variable “corolla segmentation”. The value (*V*) of each variable’s level in the index is the mean of four assigned scores.

| **Value (*V*)** | **sympetalous** | **semichoripetalous** | **choripetalous** |
| --- | --- | --- | --- |
| Score 1 | 3 | 2 | 1 |
| Score 2 | 3 | 2 | 1 |
| Score 3 | 1 | 2 | 3 |
| Score 4 | 3 | 2 | 1 |
| **Mean** | **2.5** | **2** | **1.5** |

Table S1.5. Values assigned to the levels of the floral variable “functional reproductive unit”. The value (*V*) of each variable’s level in the index is the mean of four assigned scores.

| **Value (*V*)** | **single** | **spikes/racemes** | **heads** |
| --- | --- | --- | --- |
| Score 1 | 2 | 3 | 1 |
| Score 2 | 2 | 3 | 1 |
| Score 3 | 1 | 3 | 2 |
| Score 4 | 1 | 3 | 2 |
| **Mean** | **1.5** | **3** | **1.5** |
