# Supplementary material for: Lessons from Red Data Books: Plant Vulnerability Increases with Floral Complexity
Source: PLoS One. 2015 Sep 21;10(9):e0138414. doi: 10.1371/journal.pone.0138414 (PMC4577097; doi:10.1371/journal.pone.0138414)
Supplement: S2 Text — (DOCX) [file pone.0138414.s003.docx]

**S2 Text. The effects of the individual components of the Floral Complexity Index on plant vulnerability.**

In the formulae of the GLMs corresponding to the results below, FCI was replaced by one (or all) FCI component(s). A Generalized Linear Model (GLM) was fitted in R 3.1.1 using the ‘glm’ function with a binomial probability distribution, in order to explore the relation of plant vulnerability and the independent intrinsic and extrinsic variables. A stepwise backward model selection procedure using the Akaike Information Criterion (AIC) was performed to select the best model. All independent variables were checked for collinearity before regression analysis. The final model was constructed on the basis of the AIC. As is the case with binary traits in logistic GLM, goodness-of-fit in the final models was tested graphically with validation plots of the empirical probabilities with their standard errors (see Materials and Methods).

**1. Bivariate analysis**

Results of the effects of each FCI component on plant vulnerability.

| **FCI component** | **LR *χ*^2^** | **Df** | ***P-*value** |
| --- | --- | --- | --- |
| Floral depth | 2.896 | 2 | 0.235 |
| Floral shape | 13.351 | 10 | 0.205 |
| Floral symmetry | 3.243 | 1 | 0.072 |
| Functional reproductive unit | 4.558 | 2 | 0.102 |
| Corolla segmentation | 0.691 | 2 | 0.708 |

It should be highlighted that when the effect of FCI on plant vulnerability was tested bivariately, there was a statistically significant result:

|  | **LR *χ*^2^** | **Df** | ***P-*value** |
| --- | --- | --- | --- |
| FCI | 4.065 | 1 | 0.044 |

**2. Multivariate analysis (vulnerability ~ all FCI components)**

When all the components were tested simultaneously against plant vulnerability, no combination of them was found to have a significant effect. Below, the AICs of the backward stepwise model selection process after the removal of each FCI component are presented.

| **FCI component removed** | **AIC** |
| --- | --- |
| Functional reproductive unit | 467.94 |
| Floral depth | 468.38 |
| Corolla segmentation | 471.00 |
| Floral shape | 474.26 |
| none | 484.25 |

The best fitting model (AIC: 467.94) included only floral symmetry, which was shown to have no statistically significant effect on vulnerability:

| **FCI component** | **LR *χ*^2^** | **Df** | ***P-*value** |
| --- | --- | --- | --- |
| Floral symmetry | 3.243 | 1 | 0.072 |

**3. Multivariate analysis – full model (vulnerability ~ single FCI component + extrinsic + intrinsic factors)**

Results of the best fitting GLMs (based on AIC) showing the effects of the individual FCI components on plant vulnerability. The full model includes as independent variables all the intrinsic and extrinsic factors described in Materials and Methods, apart from FCI. Each line of the table corresponds to a different best fitting GLM, where FCI was replaced by a single FCI component.

| **FCI component** | **included in the best fitting GLM** | **LR *χ*^2^** | **Df** | ***P-*value** |
| --- | --- | --- | --- | --- |
| Floral depth | no | - | - | - |
| Floral shape | no | - | - | - |
| Floral symmetry | yes | 8.549 | 1 | **0.003** |
| Functional reproductive unit | yes | 6.544 | 2 | **0.038** |
| Corolla segmentation | no | - | - | - |

**4. Multivariate analysis – full model (vulnerability ~ all FCI components + extrinsic + intrinsic factors)**

In the table below, the AICs of the backward stepwise model selection process after the removal of each FCI component are presented.

| **FCI component removed** | **AIC** |
| --- | --- |
| Corolla segmentation | 446.20 |
| Floral depth | 450.00 |
| Floral shape | 455.75 |
| none | 472.42 |

The best fitting model included both floral symmetry and functional reproductive unit, but only the first was shown to have a statistically significant effect on vulnerability:

| **FCI component** | **LR *χ*^2^** | **Df** | ***P-*value** |
| --- | --- | --- | --- |
| Floral symmetry | 7.488 | 1 | **0.006** |
| Functional reproductive unit | 5.090 | 2 | 0.078 |

**5. Goodness-of-fit of the full models including either FCI or floral symmetry (raw variable)**

In order to find the best model we compared the values of AIC and the Bayesian Information Criterion (BIC) of two full models including either FCI or floral symmetry.

| **Covariates** | **df** | **AIC** | **BIC** |
| --- | --- | --- | --- |
| *FCI* + life form + fl. color + habitat + alt. min + max. distance | 17 | 451.358 | 520.324 |
| *Fl. symmetry* + life form + fl. color + habitat + alt. min + max. distance | 17 | 451.584 | 520.549 |
