# Supplementary material for: Lessons from Red Data Books: Plant Vulnerability Increases with Floral Complexity
Source: PLoS One. 2015 Sep 21;10(9):e0138414. doi: 10.1371/journal.pone.0138414 (PMC4577097; doi:10.1371/journal.pone.0138414)
Supplement: S3 Text — (DOCX) [file pone.0138414.s004.docx]

**S3 Text**. **Results of the tests for phylogenetic signal in the Greek rare and threatened plants’ vulnerability as a raw variable (binary trait) and in its residuals in the best fitted GLM according to AIC.**

See Methods for details on each metric.

1. **Plant vulnerability –the raw variable**

***D* statistic for binary traits**

Number of permutations: 1000

Estimated *D*: 0.950

Probability of *D* resulting from no (random) phylogenetic structure: 0.135

Probability of *D* resulting from Brownian phylogenetic structure: 0

*Interpretation: D* is equal to unity if the observed binary trait has a phylogenetically random distribution across the tips (taxa) of the phylogeny, and to zero if the observed trait is as clumped as if it had evolved by Brownian motion (see [1]). In the case of plant vulnerability (Fig. S3.1), *D* shows no phylogenetic signal.


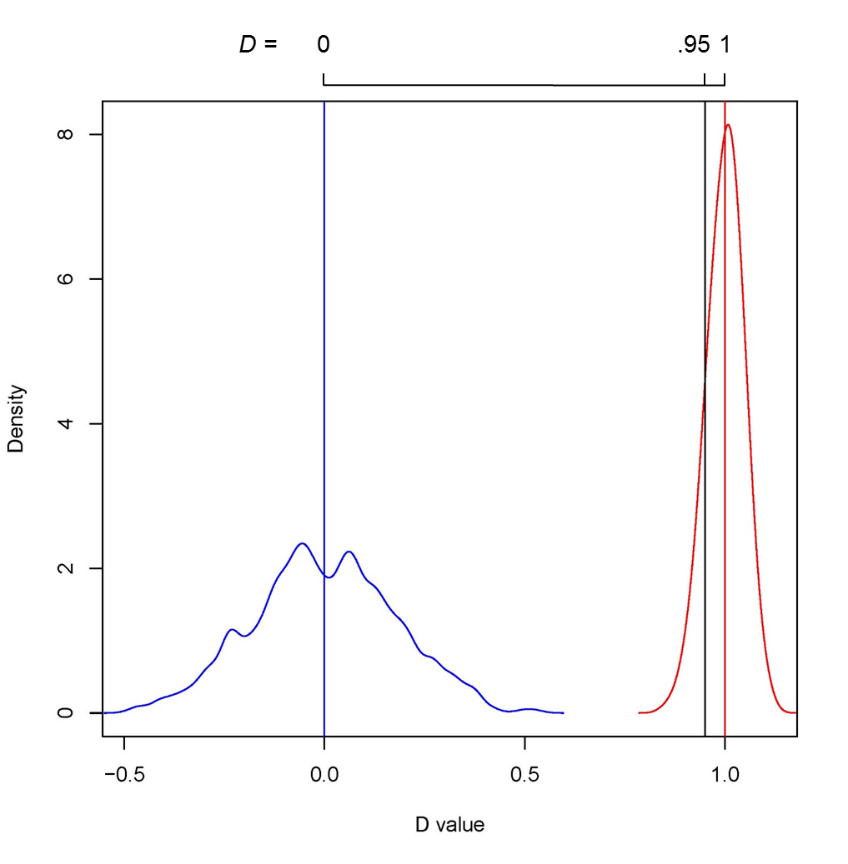


**Fig. S3.1.** Scaling of the observed *D* values (black line) for plant vulnerabity from the distributions of simulated sums of vulnerability change under models of random association (red line) and a Brownian process (blue line) (for details see [2]).

1. **Residuals of plant vulnerability**
   1. **Blomberg’s K**

| K statistic | 0.072 |
| --- | --- |
| *P*-value^1^ | 0.863 |

^1^Based on variance of phylogenetically independent contrasts, relative to tip shuffling randomizations (999 reps)

*Interpretation*: K varies continuously from zero (no phylogenetic signal) to ∞. If K = 1, then the continuous trait examined varies among the species as if it had evolved under a Brownian motion model. *P-*value <.05 would indicate that K is significantly different from zero, which is not the case for the residuals of plant vulnerability in our final GLM.

- 1. **Pagel’s λ**

| **λ** statistic^2^ | 0.055 |
| --- | --- |
| lower bound | 0.000, *p* = 0.536 |
| upper bound | 1.000, *p* = < 2.22e-16 |

^2^Acquired with likelihood ratio tests

*Interpretation:* When λ = 0, there is no phylogenetic signal, whereas λ = 1 indicates a Brownian model of evolution of the trait examined. Lower bound gives the *p*-value when maximum likelihood (ML) λ is compared to λ = 0, upper bound gives the *p*-value when ML λ is compared to λ = 1. Plant vulnerability has practically zero probability of showing a phylogenetic signal according to Brownian evolutionary model.

**References**

Fritz SA, Purvis A. Selectivity in mammalian extinction risk and threat types: a new measure of phylogenetic signal strength in binary traits. Conserv Biol. 2010;24:1042–1051.

Orme D. The caper package: comparative analysis of phylogenetics and evolution in R. The Comprehensive R Archive Network, Packages; 2013. Available at <http://cran.r-project.org/web/packages/caper/vignettes/caper.pdf>
